# Design of nucleotide-mimetic and non-nucleotide inhibitors of the translation initiation factor eIF4E: Synthesis, structural and functional characterisation

**DOI:** 10.1016/j.ejmech.2016.08.047

**Published:** 2016-11-29

**Authors:** Fadi Soukarieh, Matthew W. Nowicki, Amandine Bastide, Tuija Pöyry, Carolyn Jones, Kate Dudek, Geetanjali Patwardhan, François Meullenet, Neil J. Oldham, Malcolm D. Walkinshaw, Anne E. Willis, Peter M. Fischer

**Affiliations:** a School of Pharmacy and Centre for Biomolecular Sciences, University of Nottingham, University Park, Nottingham NG7 2RD, UK; b Centre for Translational Chemical Biology, University of Edinburgh, Michael Swann Building, King's Buildings, Mayfield Road, Edinburgh EH9 3JR, UK; c School of Chemistry, University of Nottingham, University Park, Nottingham NG7 2RD, UK; d M.R.C. Toxicology Unit, University of Leicester, Lancaster Road, Leicester LE1 9HN, UK

**Keywords:** Cancer, eIF4E, Protein synthesis, mRNA translation, Cap-binding inhibitor, eIF, eukaryotic translation initiation factor, 4E-BP, eF4E-binding protein, FP, fluorescence polarisation, m^7^G, N^7^-methyl guanosine, RRL, rabbit reticulocyte lysate

## Abstract

Eukaryotic translation initiation factor 4E (eIF4E) is considered as the corner stone in the cap-dependent translation initiation machinery. Its role is to recruit mRNA to the ribosome through recognition of the 5′-terminal mRNA cap structure (m^7^GpppN, where G is guanosine, N is any nucleotide). eIF4E is implicated in cell transformation, tumourigenesis, and angiogenesis by facilitating translation of oncogenic mRNAs; it is thus regarded as an attractive anticancer drug target. We have used two approaches to design cap-binding inhibitors of eIF4E by modifying the N^7^-substituent of m^7^GMP and replacing the phosphate group with isosteres such as squaramides, sulfonamides, and tetrazoles, as well as by structure-based virtual screening aimed at identifying non-nucleotide cap-binding antagonists. Phosphomimetic nucleotide derivatives and highly ranking virtual hits were evaluated in a series of *in vitro* and cell-based assays to identify the first non-nucleotide eIF4E cap-binding inhibitor with activities in cell-based assays, *N*-[(5,6-dihydro-6-oxo-1,3-dioxolo[4,5-*g*]quinolin-7-yl)methyl]-*N*′-(2-methyl-propyl)-*N*-(phenyl-methyl)thiourea (**14**), including down-regulation of oncogenic proteins and suppression of RNA incorporation into polysomes. Although we did not observe cellular activity with any of our modified m^7^GMP phosphate isostere compounds, we obtained X-ray crystallography structures of three such compounds in complex with eIF4E, 5′-deoxy*-*5′-(1,2-dioxo-3-hydroxycyclobut-3-en-4-yl)amino-*N*^7^-methyl-guanosine (**4a**), *N*^7^-3-chlorobenzyl-5′-deoxy*-*5′-(1,2-dioxo-3-hydroxy-cyclobut-3-en-4-yl)amino-guanosine (**4f**), and *N*^7^-benzyl-5′-deoxy*-*5′-(trifluoromethyl-sulfamoyl)guanosine (**7a**). Collectively, the data we present on structure-based design of eIF4E cap-binding inhibitors should facilitate the optimisation of such compounds as potential anticancer agents.

## Graphical abstract

**Figure:**
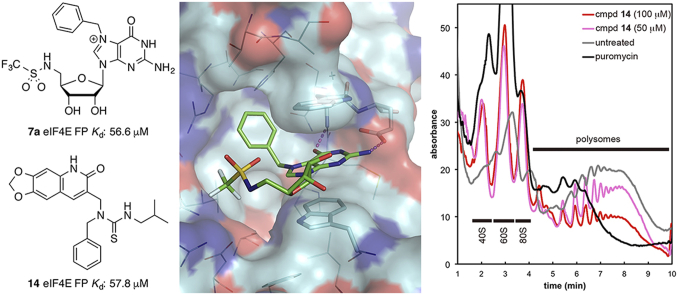

## 1 Introduction

In eukaryotes, most mRNAs are translated using a cap-dependent mechanism, which consists of three stages: initiation, elongation, and termination. The initiation step requires a number of eukaryotic translation initiation factors (eIFs) [1]. Initially, the 40S ribosomal subunit associates with Met-tRNAi and a group of initiation factors, including eIF1, eIF1A, eIF2, eIF3, and eIF5 to form the 43S pre-initiation complex [2]. This complex is then recruited to the 5′-end of capped mRNA by another complex of factors (known as eIF4F) consisting of eIF4E, eIF4G, and eIF4A. eIF4E is an indispensable element for cap-dependent translation initiation and plays a major role in recognition of the mRNA cap structure (m^7^GpppN, where N is any nucleotide). It binds to eIF4G, which serves as a scaffolding protein to gather eIF4A (ATP-dependent RNA helicase), eIF4B, eIF3, and poly-A-binding protein to form the 48S complex [3], [4]. This complex then scans the mRNA for an initiation codon and, once this is located, the 60S ribosomal subunit joins to form the elongation-competent fully functional 80S.

Under typical circumstances, eIF4E is the least abundant of the cellular translation initiation components [5] and it represents the rate-limiting element for the initiation process [6], [7]. Under resting conditions, eIF4E is kept in an inactive form bound to 4E-binding proteins (4E-BPs); once the latter have been phosphorylated through the PI3K-AKT-mTOR signalling pathway, eIF4E is released and can associate with eIF4G to launch translation.

eIF4E is overexpressed in numerous human tumours, and it contributes to transformation, tumourigenesis, and progression of cancers [8], [9]. eIF4E overexpression facilitates translation of weak and highly structured mRNAs that typically encode proteins involved in cancer pathology, such as proto-oncoproteins (*e.g.* cyclin D1, ornithine decarboxylase), angiogenesis factors (*e.g.* FGF-2, VEGF), and factors related to tumour invasiveness, such as MMP-9 [10], due to the ability of eIF4E to stimulate eIF4A [11]. For these reasons, eIF4E represents an attractive cancer drug target [12]. Moreover, a recent study suggests that targeting eIF4E for cancer treatment has minimal effects on growth of –and protein synthesis in– healthy cells [13].

**Fig. 1:**
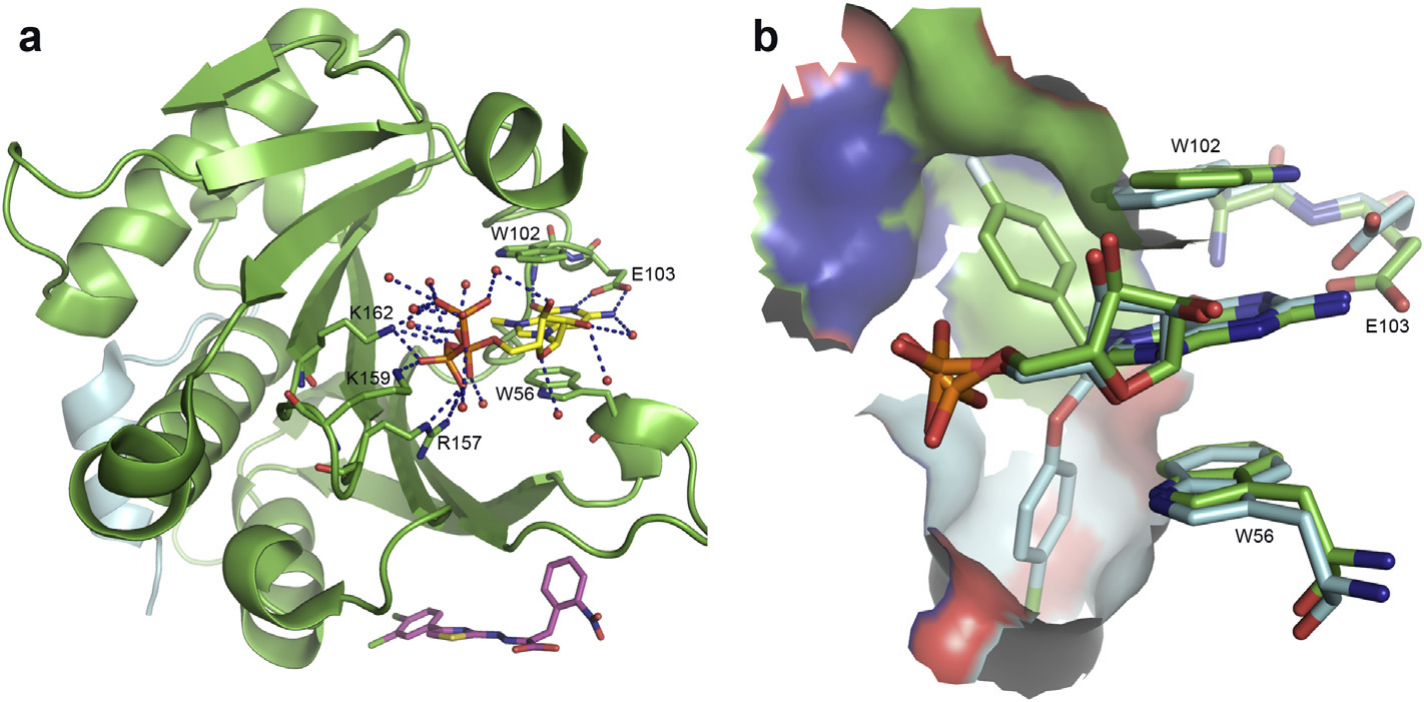
(**a**) mRNA cap recognition (represented by m^7^GTP shown as yellow CPK sticks) and binding of eIF4G or 4E-BPs (represented by a peptide derived from 4E-BP1 shown as a cyan cartoon) occurs on opposite faces of eIF4E (green cartoon). Apart from direct cap-binding antagonists, allosteric inhibitors (binding pose of 4-EGI1 [22] shown as magenta sticks) and inhibitors derived from eIF4G and 4E-BPs [23] are being developed. Whereas m^7^GTP-binding is dominated by polar interactions between the cationic N-methylpurine system and eIF4E residues W56, W102, and E103 (cation–π interaction and H-bonds), as well as the phosphate groups with residues R157, K159, and K162, (**b**) GMP derivatives with N^7^-substituents other than methyl, such as 4-fluorobenzyl [24] (green) or (4-chlorophenoxy)ethyl [20] (cyan), also make hydrophobic contacts with two concave lipophilic pockets (surface representations) behind the W56—W102 stack. Figure constructed from PDB entries 2V8W, 2V8Y, 4DT6, and 4TPW. 3D-Structure illustrations in this and subsequent Figures were prepared using MacPyMOL (The PyMOL Molecular Graphics System, Version 1.2, Schrödinger, LLC). (For interpretation of the references to colour in this figure legend, the reader is referred to the web version of this article.)

Strategies to design eIF4E–mRNA cap-binding antagonists have been based on nucleoside (purine N^7^-substitutions) [14], [15], [16], [17] and nucleotide (altered phosphate groups) [17], [18], [19] modifications of 7-methylguanosine (m^7^G) nucleotides (Fig. 1) [12], including recent work affording for the first time m^7^G monophosphate (m^7^GMP) nucleotide mimetic compounds with high affinity for eIF4E [20]. However, to date no cell-permeable small-molecule eIF4E cap-binding antagonists have been reported. As with antiviral agents derived from nucleotides, prodrugs that mask the ionic nature of phosphate groups, such as phosphate esters of phosphoramidates, may offer an avenue for the design of cell-permeable eIF4E inhibitors, but this strategy has not progressed very far as yet [21].

Here we report on new methods to design cap antagonists and we show a group of nucleotide mimetic compounds with phosphate group isosteres and various purine N^7^-substituents. The biological activities of these compounds were assessed using a range of techniques and the eIF4E-binding modes of three compounds were determined experimentally using X-ray crystallography. However, despite phosphate group modifications, these nucleotide mimetics still exhibit poor cellular bioactivity. Therefore, a computer-aided drug design method was exploited to design a set of non-nucleotide compounds to provide the first small-molecule eIF4E inhibitor possessing cellular activity consistent with blocking of eIF4E-mediated initiation of translation.

## 2 Results and discussion

### 2.1 Design and synthesis of nucleotide monophosphate mimetic eIF4E inhibitors

One of the main challenges in the design of inhibitors of eIF4E cap binding has been to achieve membrane permeability and thus cellular bioavailability [20]. This is due to the fact that the affinity of eIF4E for the RNA m^7^GpppN cap structure derives in large part from polar interactions between the ligand triphosphate group and the receptor protein (Fig. 1a) [15], [25]. In order to address the permeability problem, as well as the intrinsic hydrolytic and enzymatic lability of nucleotides, we aimed to design nucleoside monophosphate mimetics [25], [26]. Several phosphate group replacements were investigated, such as squaramides, sulfonamides, and tetrazoles. Since *e.g.* m^7^GMP has much lower affinity for eIF4E than m^7^GTP [25], we expected to compensate the loss in affinity with nucleoside monophosphate mimetics through optimal purine N^7^-substituent modifications, based on previous studies demonstrating that replacing the N^7^-methyl group with bulkier groups contributed significantly to ligand affinity (Fig. 1b) [27], [28].

Squaric acid possesses similar charge distribution, polarity, and acidic properties as phosphoric acid, hence the squaramide group could represent an isostere for the phosphate group [29], [30]. Similarly, sulfonamide derivatives have been reported as phosphate mimics in the design of tyrosine phosphatase inhibitors [31]. The third candidate we considered for phosphate group replacement was a tetrazole [32], as this system mimics the acidic features of the phosphate group, provided the proton attached to one of the tetrazole ring nitrogens is not replaced [33].

**Scheme 1:**
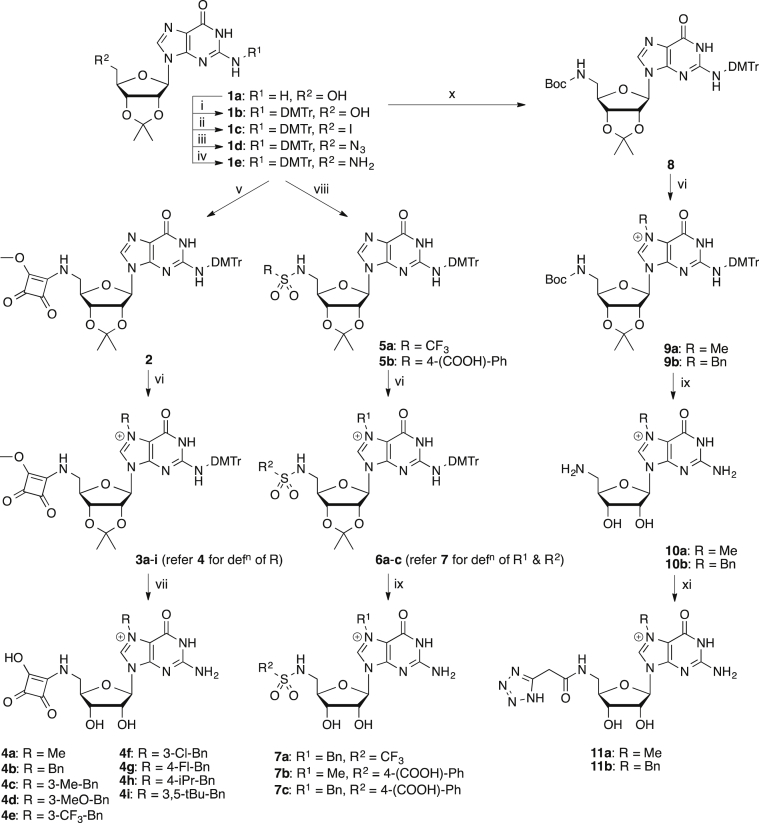
Preparation of nucleotide mimetics **4a**–**4i**, **7a**–**7c**, and **11a**–**11b**^*a*^. ^*a*^ Conditions: (i) DMTr-Cl, Me_3_SiCl; (ii) (PhO)_3_P^+^MeI^−^, THF, −78 °C; (iii) NaN_3_, DMF, 85 °C; (iv) Ph_3_P, pyridine, NH_4_OH; (v) dimethyl squarate, iPr_2_NEt, MeOH; (vi) R-X, DMF; (vii) NaI, Me_2_CO, Δ, then 80% aq HCOOH, 24 h; (viii) RSO_2_Cl, Et_3_N, CH_2_Cl_2_; (ix) 80% aq HCOOH, 12 h; (x) Boc_2_O, Et_3_N, CH_2_Cl_2_, 24 h; (xi) 5-tetrazole acetic acid, DIC, Et_3_N, DMF.

The key synthetic precursor for the preparation of our nucleotide cap mimetics was the protected guanosine derivative **1e** (Scheme 1) [34], which was prepared by first blocking the 2′,3′-vicinal glycol of guanosine as the acetonide **1a** [35], followed by dimethoxytritylation of the exocyclic amine group (**1b**) [36], and transformation of the 5′-hydroxyl group to the amine *via* successive conversion to the iodide **1c** and azide **1d**, and reduction of the latter [37]. The amine **1e** was condensed either with dimethyl squarate [29] to afford the squaramide derivative **2** or with polar sulfonyl chlorides to furnish the sulfonamide derivatives **5**. These intermediates were N^7^-alkylated using a range of alkyl or aryl bromides [14] to provide the methyl squaramides **3** and sulfonamides **6**. The methyl squaramide groups of the former were first demethylated with sodium iodide in acetone [38], followed by treatment with aqueous formic acid to afford the squaramides **4**. The acetonide group of precursors **6** was removed similarly with formic acid to provide the sulfonamides **7** [39]. The tetrazole derivatives **11** were also elaborated from **1e**, by Boc-protection of the 5′-amino group (**8**), followed by N^7^-alkylation (**9**), global deprotection (**10**), and acylation of the 5′-amino group with 1*H*-tetrazole-5-acetic acid.

### 2.2 Design of non-nucleotide eIF4E inhibitors

As an alternative to nucleoside monophosphate mimetics we considered structure-based design of eIF4E cap-binding antagonists. For this purpose we docked multi-conformer libraries of lead-like molecules from the ZINC [40] database to a model of the Bn^7^GMP-binding site of eIF4E derived from a complex crystal structure we reported earlier (PDB entry 2V8Y) [15] using shape-based fast rigid exhaustive docking (FRED) [41], [42]. We applied a combination of scoring functions, including functions based on Poisson-Boltzmann electrostatic potentials, in order to ensure that interactions of virtual hits mimicking the cation–π interaction [43], [44] of the N^7^-methylguanine system in the mRNA cap with the eIF4E W56—W102 indole side chains (Fig. 1a) would be rewarded. The virtual screening protocol was validated by docking and scoring a set of 500 randomly-chosen diverse decoy molecules from the ZINC database spiked with a set of N^7^-alkylated cap-derived nucleotides known to bind eIF4E, as well their non-alkylated counterparts, which are known not to interact with eIF4E [25]. This analysis revealed that the N^7^-alkylated cap-derived nucleotides populated the top 12% of the hit list from the combined scoring protocol. Based on this result we proceeded to screen the entire lead-like subset of the ZINC database. We acquired several hundred of the top-ranking virtual hits and submitted these to the mass spectrometry-based assay described earlier [45], as well as the fluorescence polarisation (FP) assay described below. The chemical structure and predicted properties of the most highly scored virtual hits are shown in Table 1.

### 2.3 Biological activity of nucleotide monophosphate mimetic and non-nucleotide eIF4E inhibitors

**Fig. 2:**
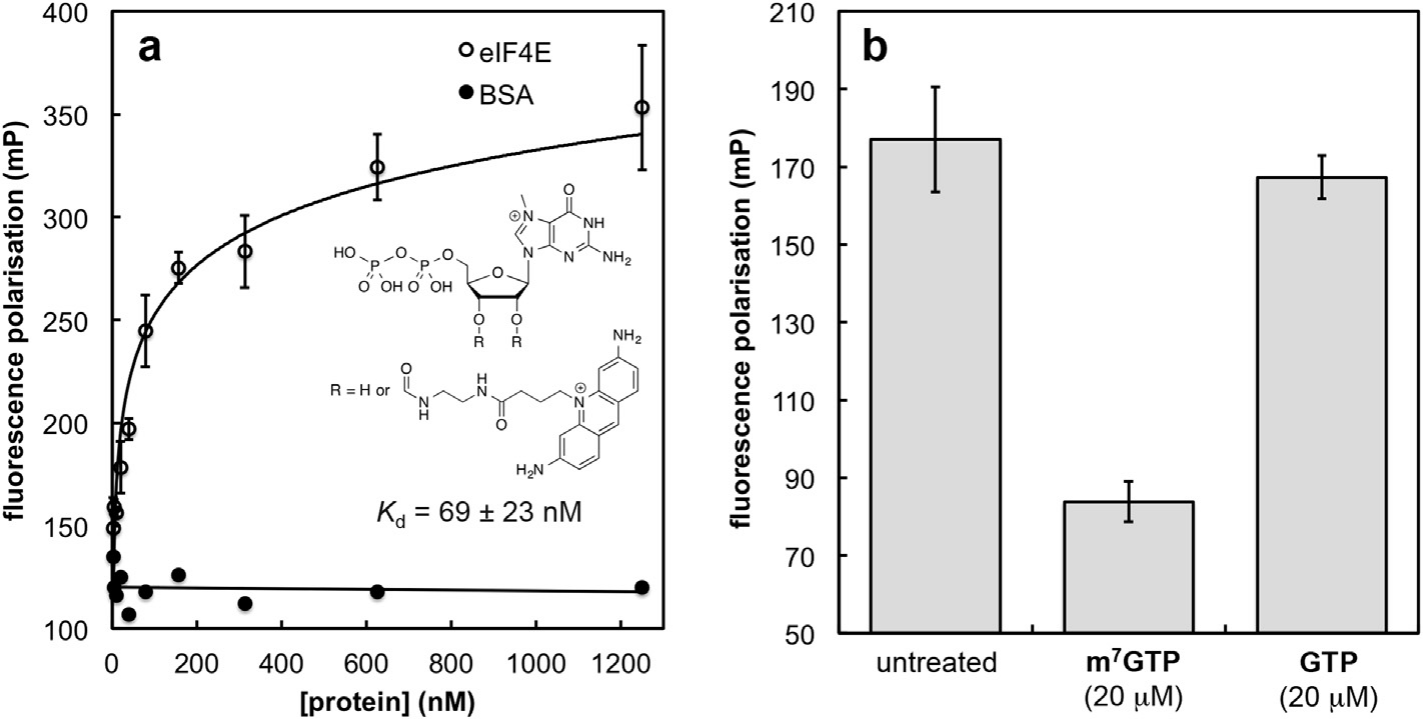
(**a**) Recombinant eIF4E or bovine serum albumin (BSA) was titrated against the FP probe ATTO-465-labelled 2′/3′-*O*-(2-aminoethylcarbamoyl)-7-methyl-guanosine-5′-diphosphate (EDA-m^7^GDP-ATTO-465, structure shown inset; 10 nM) and a *K*_d_ value with respect to eIF4E binding was calculated. (**b**) The FP assay is specific for the unique structural feature of the mRNA cap and can distinguish between m^7^GTP and GTP (eIF4E concentration: 90 nM).

In order to assess our m^7^GMP-derived compounds and virtual screening hits as mRNA cap-binding antagonists, we developed an FP assay (Fig. 2) based on displacement of a fluorescently labelled m^7^GDP derivative from eIF4E by cap-binding inhibitors. Assay optimisation showed that a tracer concentration of 10 nM and an eIF4E concentration of 90 nM was optimal. All test compounds were screened initially at 100 μM and actives were titrated to calculate *K*_d_ values, summarised in Table 2.

As expected, m^7^GTP had almost 10-fold higher affinity for eIF4E in our FP assay than m^7^GMP. Although the majority of the squaramide (**4**), sulfonamide (**7**), and tetrazole (**11**) phosphate-mimetic analogues inhibited tracer binding in the FP assay at the prescreen concentration of 100 μM to some extent, only the sulfonamides **7a** and **7b** showed levels of inhibition that permitted construction of a sufficiently wide dose–response titration for accurate *K*_d_ determination. Of the squaramides, all derivatives except **4a**, **4c**, and **4f** showed insignificant inhibition (<10%) in the prescreen. Surprisingly, in the context of the squaramide phosphomimetic group, the expected enhancement of affinity upon replacing the N^7^-methyl group (**4a**) with a benzyl group (**4b**) was not observed. Furthermore, substitution of the phenyl group with small and electronically neutral groups (3-Me, **4c**; 3-Cl, **4f**) retained the modest activity of **4a** and **4b**, whereas introduction of larger and more polar substituents reduced or abolished activity. The trifluoromethyl- and the *para*-carboxyphenyl sulfonamides **7a** and **7b** were the only nucleoside monophosphate mimetic compounds for which *K*_d_ values could be obtained, and these are significantly higher than that of m^7^GMP. The tetrazole derivatives **11** had very modest activity. Here the N^7^-benzyl derivative **11b** was somewhat more active than the N^7^-methyl compound **11a**.

Amongst the virtual screening hits, a cluster of 4,5-bisaryl-2-carboxymethylthio-1,2,3-triazoles **12a**–**c** was identified but these showed low activity in the FP prescreen and *K*_d_ values could not be determined. Of the somewhat structurally related quinolinones **13** and **14**, the former was essentially without activity in the FP assay, whereas the latter was clearly active, at a level similar to the best of the nucleotide derivatives (**7b**). The thiazolone **15** and the pyridothiazolopyrimidine **16** showed no activity in the FP assay.

**Fig. 3:**
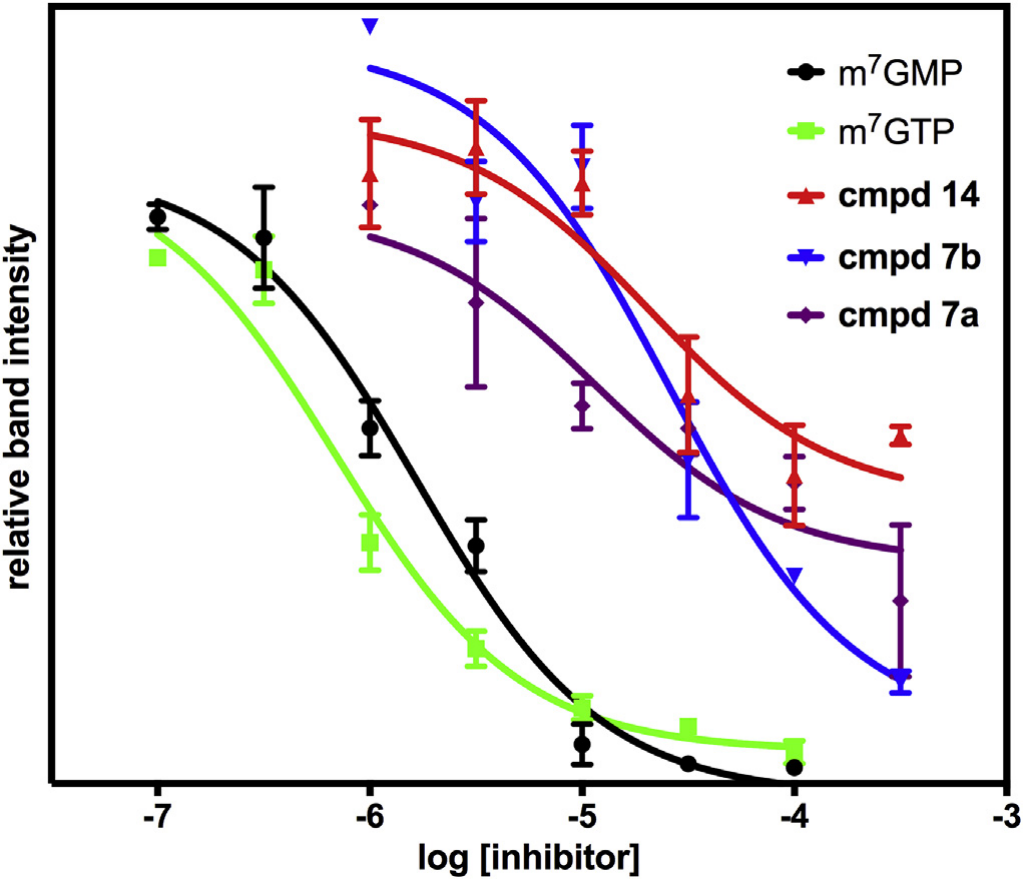
Dose–response curves for m^7^GMP, m^7^GTP, as well as compounds **7a**, **7b**, and **14** in the radiolabelled and capped RNA (5 nM) – eIF4E (1.5 μM) UV cross-linking assay. Inhibitor concentrations are plotted against autoradiography band intensities following separation of the cross-linked RNA–eIF4E complexes.

Further to confirm inhibition of eIF4E cap binding, a radiometric competition binding assay was developed based on an *in vitro* UV cross-linking technique widely used to study protein–RNA interactions [48]. In this assay, a radiolabelled and capped RNA was incubated with eIF4E in the presence or absence of putative inhibitors **4**–**16**. The RNA–eIF4E complexes formed were cross-linked, separated by SDS-PAGE, and quantitated by autoradiography. The assay was validated with positive controls (m^7^GMP and m^7^GTP), test compounds were pre-screened at 100 μM (Table 2), and for sufficiently active compounds half-maximal inhibitory concentration (IC_50_) values were determined (Fig. 3).

As expected, m^7^GMP and m^7^GTP displayed similar activity levels in the UV cross-linking assay as in the FP assays. The squaramides **4** were inactive with the exception of the derivative **4h**. All three sulfonamides **7** showed activity in the UV cross-linking assay and for **7a** and **7b** IC_50_ values could be determine (Fig. 3). For the inhibitors from the virtual screen (**11**–**16**), the UV cross-linking assay results mirrored those from the FP assay but an IC_50_ value could only be determined for quinolinone **14**.

**Fig. 4:**
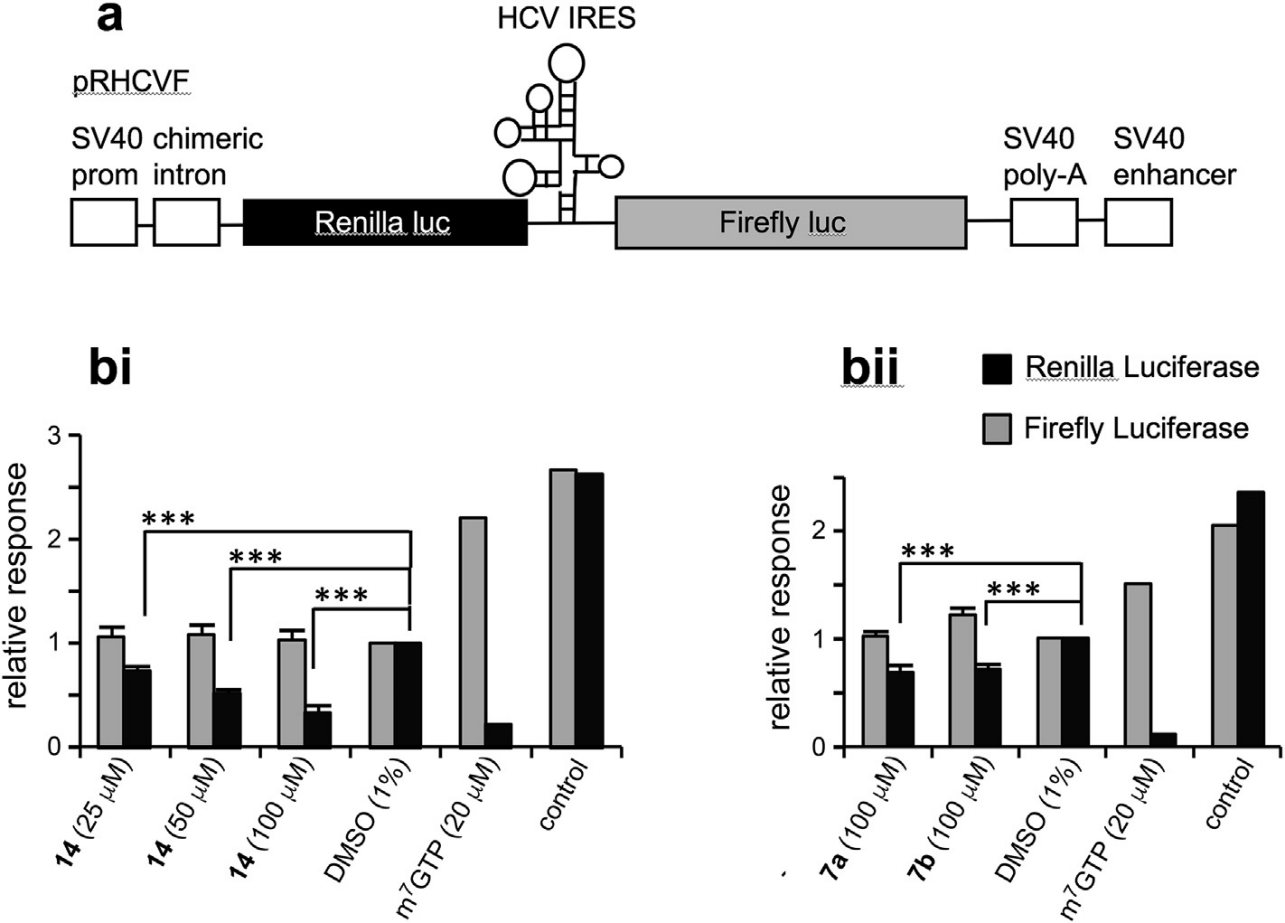
Compounds **7a**, **7b**, and **14** inhibit cap-dependent translation, but not cap-independent translation driven by the HCV IRES: (**a**) Schematic of the construct containing the HCV IRES. Cap-dependent translation drives expression from the *Renilla* luciferase transcript, whereas cap-independent translation drives protein synthesis from the *Firefly* luciferase transcript. (**b**) Rabbit reticulocyte lysates were primed with pRHCVF RNA and treated with compound **14** (**i**) or **7a** or **7b** (**ii**) at the doses shown. The compounds were dissolved in DMSO, which is known to inhibit the reticulocyte lysate system when compared to the control without DMSO. Therefore a 1% solution of DMSO was used as an additional control and the degree of inhibition measured relative to this value. Experiments were performed on three independent occasions, significance (***P < 0.001) was calculated using Student's t-test (error bars S.D.). Black bars denote *Renilla* luciferase and grey bars *Firefly* luciferase.

For the nucleoside monophosphate mimetic compounds, which we expected to be devoid of cellular bioactivity, we also sought to obtain a functional read-out of eIF4E inhibition. For this purpose we employed a cell-free rabbit reticulocyte lysate (RRL) system to observe the translational consequences of eIF4E inhibition [49]. Test compounds m^7^GMP, m^7^GTP, as well as the sulfonamides **7a** and **7b** were incubated in the RRL system, which was primed with luciferase RNA, and expression of luciferase was then quantitated. From the results (Table 2) it can be observed that all four test compounds inhibited luciferase synthesis. For m^7^GMP and m^7^GTP the IC_50_ values in the RRL assay were significantly higher than those determined for competitive binding, perhaps due to limited stability of these nucleotides in RRL, whereas the drop-off in activity was much less pronounced for sulfonamides **7a** and **7b**. RRLs were then primed with bicistronic mRNAs where the initiation of translation of the upstream *Renilla* cistron occurs in a cap-dependent manner, while the translation of the downstream firefly luciferase cistron is driven by the HCV internal ribosome entry segment and is therefore independent of the cap structure at the 5′ end of the mRNA (Fig. 4). This bicistronic RNA allowed the comparison of the effect of quinonlinone **14** and the *para*-carboxyphenyl sulfonamides **7a** and **7b** on cap-dependent and cap-independent translation simultaneously. The data show that while, as expected, there was inhibition of synthesis from the cap-dependent *Renillia* luciferase mRNA, there was no effect on the translation initiation of the firefly luciferase mediated by the HCV-IRES, suggesting that sulfonamides **7a** and **7b** and quinolinone **14** inhibit cap-dependent translation (Fig. 4).

In order to investigate the effect of the eIF4E inhibitors on overall protein synthesis in cells, we employed ^35^S-Met labelling [50] to measure the amount of newly synthesised total protein in HeLa cells after incubation for 24 h with test compounds (the protein synthesis inhibitor puromycin was used as a positive control). None of the nucleoside phosphates (m^7^GMP and m^7^GTP) or phosphate mimetics (**7a** and **7b**) showed measurable inhibition of cellular protein synthesis, indicating that membrane permeability of the nucleoside phosphate mimetic compounds, including the sulfonamides **7a** and **7b**, is still sub-optimal. Quinolinone **14**, on the other hand, which was the only virtual screening hit with activity in the UV cross-linking assay, also inhibited protein synthesis in both HeLa and MCF-7 cells with IC_50_ values similar to the activity levels observed in cell-free assays, suggesting that this compound is cell-permeable. Based on the predicted physicochemical properties of this compound (Table 2), this finding was not unexpected.

**Fig. 5:**
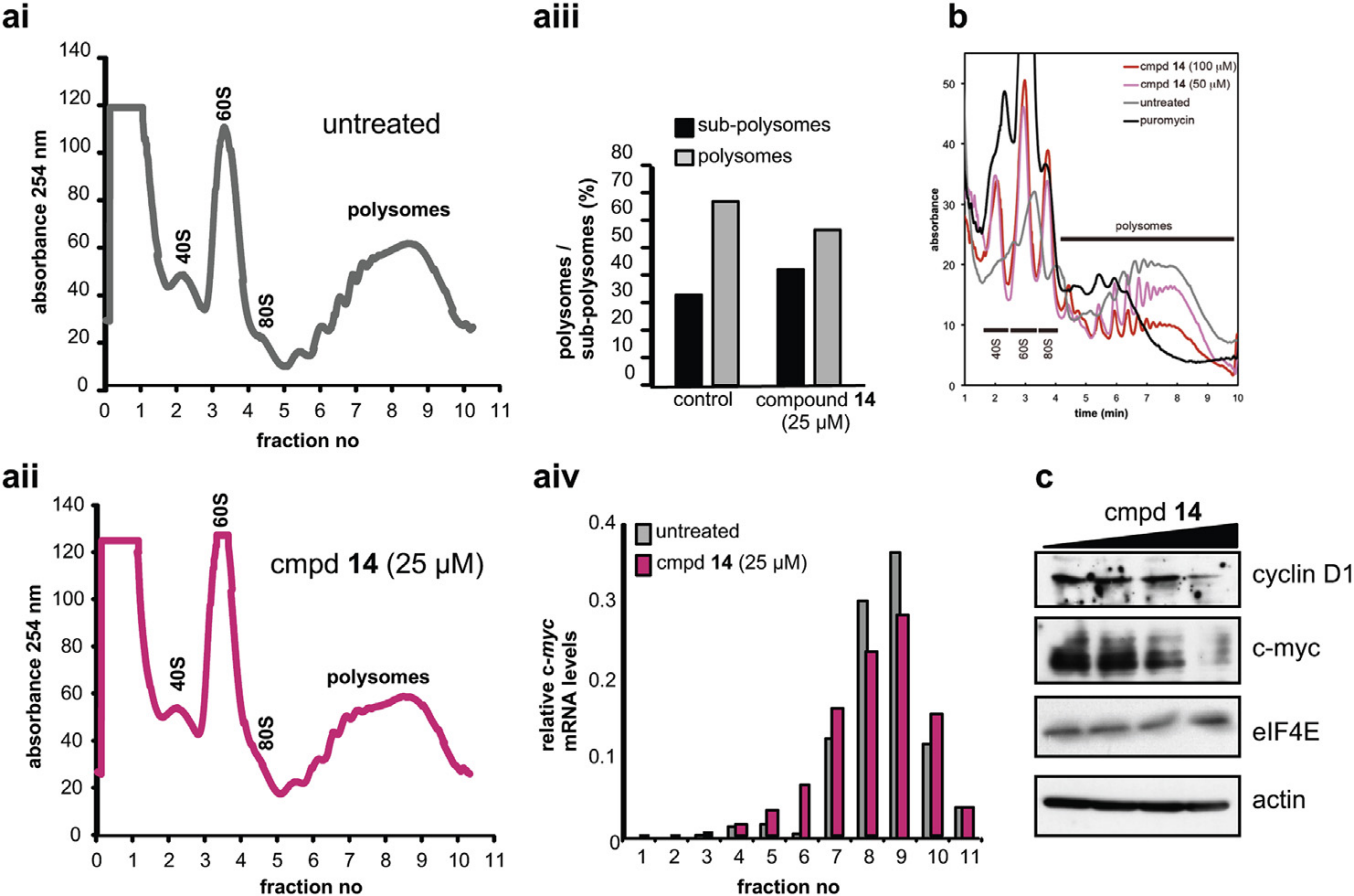
(**ai** and **aii**) Cells, either control (grey line) or treated with 25 mM compound **14** (pink line) were lysed and post-nuclear extracts were applied to 10–50% sucrose gradients to allow the separation of actively translating ribosomes (polysomes) from subpolysomal material (*e.g*. 40S and 60S ribosomal subunits). (**aiii)** Analysis of the areas under the curves attributable to the polysomes and subpolysomes in the control (**ai**) and treated (**aii**) samples showed a decrease in the amount of polysomes in treated cells and a concomitant increase in the subpolysomal fractions. (**aiv**) To examine whether there was a decrease in the amount of c-*myc* mRNA associated with the polysomes following treatment with compound **14**, RT-qPCR was performed on individual fractions. The data show that there is a shift of c-*myc* mRNA to fractions that contain fewer polysomes indicative of translational repression of this mRNA. (**b**) To examine the effect of higher doses of compound **14** on polysome formation, cells were treated with 50 μM (pink line) and 100 μM (red line) and gradients repeated. The data show a greater reduction in the amount of polysomal material under these conditions. (**c**) Western blot analyses of HeLa lysates treated for 2 h with vehicle only (DMSO) or with compound **14** (20, 50 and 100 μM). (For interpretation of the references to colour in this figure legend, the reader is referred to the web version of this article.)

For quinolinone **14**, which was observed to inhibit cellular protein synthesis, we investigated if this effect was indeed due to suppression of translation, as would be expected of an eIF4E cap-binding inhibitor. For this purpose we used polysome analysis [51]. HeLa cells were treated for 2 h with test compound **14** or puromycin as a positive control, harvested, treated with cycloheximide in order to trap RNA–ribosome complexes, and cell lysates were fractionated (Fig. 5a, i–iv). For cells treated with quinolinone **14**, there was a decrease of the amount of RNA incorporated in multiple ribosomes (polysomes) compared to untreated cells and a concomitant increase in the sub-polysomal material, consistent with a block in initiation (Fig. 5a and b), supporting our data which shows **14** to suppress cap-dependent translation in cells.

To examine whether there was an effect on the expression of proteins which are known to be eIF4E dependent and are associated with tumorigenesis, lysates of HeLa cells treated for 2 h with quinolinone **14** were probed by western blotting for c-*myc* and cyclin D1 protein, with actin used as a loading control. The data show that at 50 and 100 μM treatment concentrations of **14** there were decreases in levels of both proteins (Fig. 5c). To confirm that the decrease in protein levels corresponded with altered polysomal association, mRNAs were isolated from polysomal and sub-polysomal regions of the gradients and RT-PCR was used to assess the relative amount of c-*myc* mRNA before and after treatment. The data show that following treatment with quinolinone **14** there was a small shift of the c-*myc* mRNA into the sub-polysomes, consistent with a decrease in its translation (Fig. 5 aiv).

### 2.4 X-ray crystallography

eIF4E was co-crystallised with either an eIF4G- or a 4E-BP-derived peptide (designated 4G and 4EBP, respectively) and one of three of the m^7^GMP-derived analogues (squaramides **4a** and **4f**, sulfonamide **7a**). All complex structures showed the characteristic cupped hand structure of eIF4E [52] with the ligands occupying the cap-binding site.

**Fig. 6:**
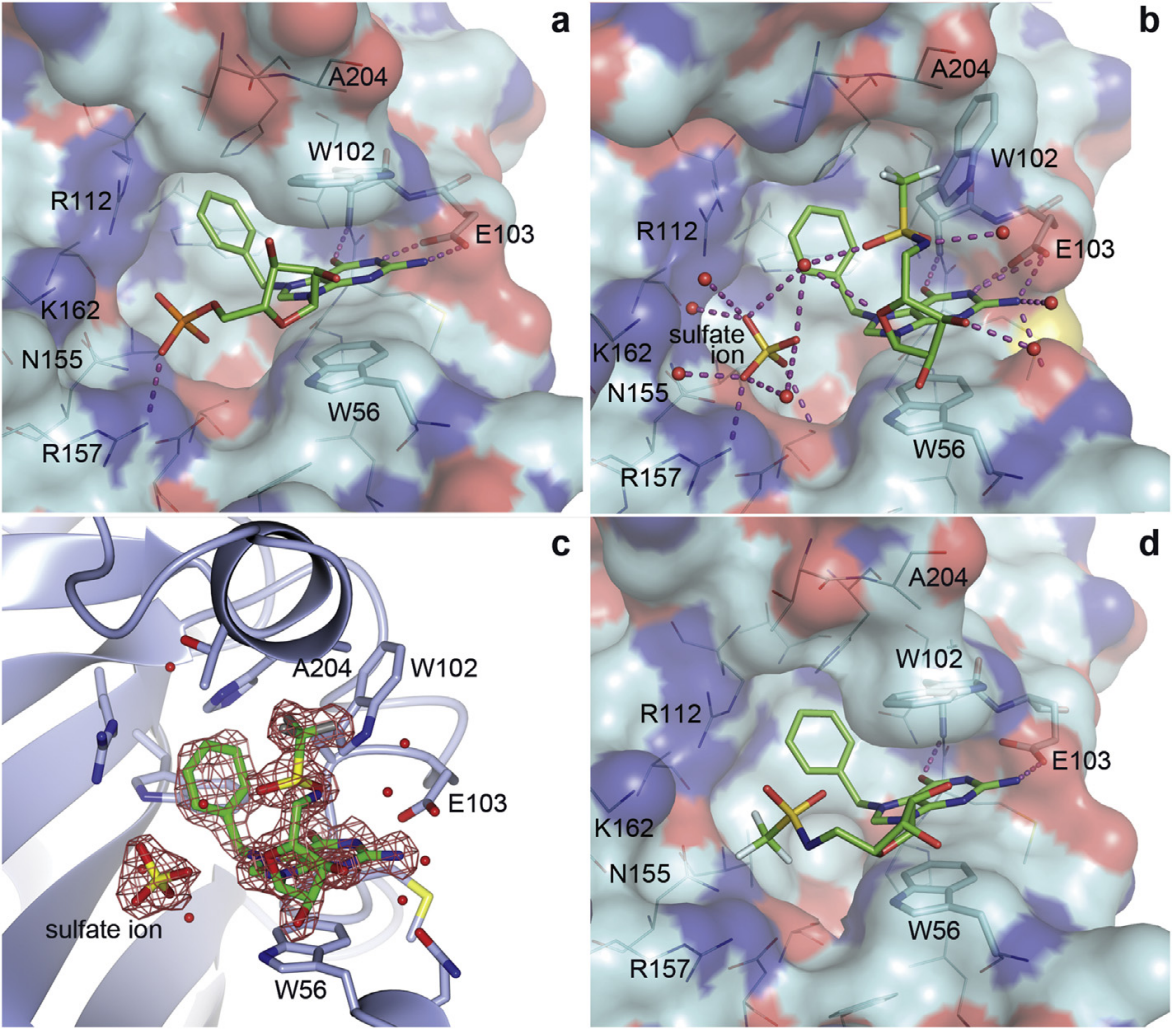
(**a**) eIF4E binding pose of Bn^7^GMP (PDB code 2V8X). (**b**) Binding pose of sulfonamide **7a**, accompanied by a sulfate ion in eIF4E (crystallographic water molecules are indicated by red spheres). (**c**) Electron density (red mesh, 0.53 electrons/Å^3^) contoured at 1.5 Å around ligand **7a** and sulfate ion. (**d**) Binding pose of **7a** following back-soaking (see text). (For interpretation of the references to colour in this figure legend, the reader is referred to the web version of this article.)

The initial eIF4E:4G:**7a** complex structure (Fig. 6b and c) was solved to a resolution of 1.71 Å (Table 3) with the ligand **7a** residing in the cap-binding site, accompanied by a single sulfate ion (from the crystallisation buffer). The guanine of **7a** makes three H-bonds, two with the side chain of E103 and one with the backbone carbonyl of W102. The sulfate ion interacts directly with eIF4E through R157 and makes additional water-bridged interactions with R112, N155, R157, and K162, as well as to the sulfonamide group of **7a**. The ligand **7a** itself interacts with four neighbouring or bridging waters. The unusual binding mode observed was unpredicted as it was expected that the guanine would make the same or similar cation-π stacking interaction as seen with all other cap-based analogues to date (compare Fig. 6a) [12], [15], [20]. However, the presence of the sulfate ion allows **7a** to ‘fold′ back on itself and the positively charged guanine forms a stabilising intramolecular electrostatic interaction with the electronegative sulfonamide group. In order to accommodate this ligand conformation, W102 is reoriented out of the binding pocket from its normal cation-π interaction position. W56 remains in the usual position, providing a stacking interaction with the guanine. In comparison to other complex structures with cap derivatives (Fig. 6a) the sulfate ion occupies the same position as the α-phosphate. Presumably the unusual bound conformation of **7a** observed was favoured due to high sulfate concentration in the crystallisation buffer.

In order to test this hypothesis, a crystal from these conditions was transferred to an identical crystallisation drop but lacking ammonium sulfate. The structure from this crystal was solved to 2.69 Å (Table 3) and shows that there is mixed occupancy for the ligand. The occupancies for both conformations are almost equal (52:48) with the higher occupancy conformation being one where the trifluoromethylsulfonamide group occupies the α-phosphate binding position (Fig. 6d), previously occupied by sulfate. As expected, the displacement of the sulfate was accompanied by repositioning of W102 to form the usual cation-π ‘sandwich′ interaction with the positively charged guanine moiety and W56.

**Fig. 7:**
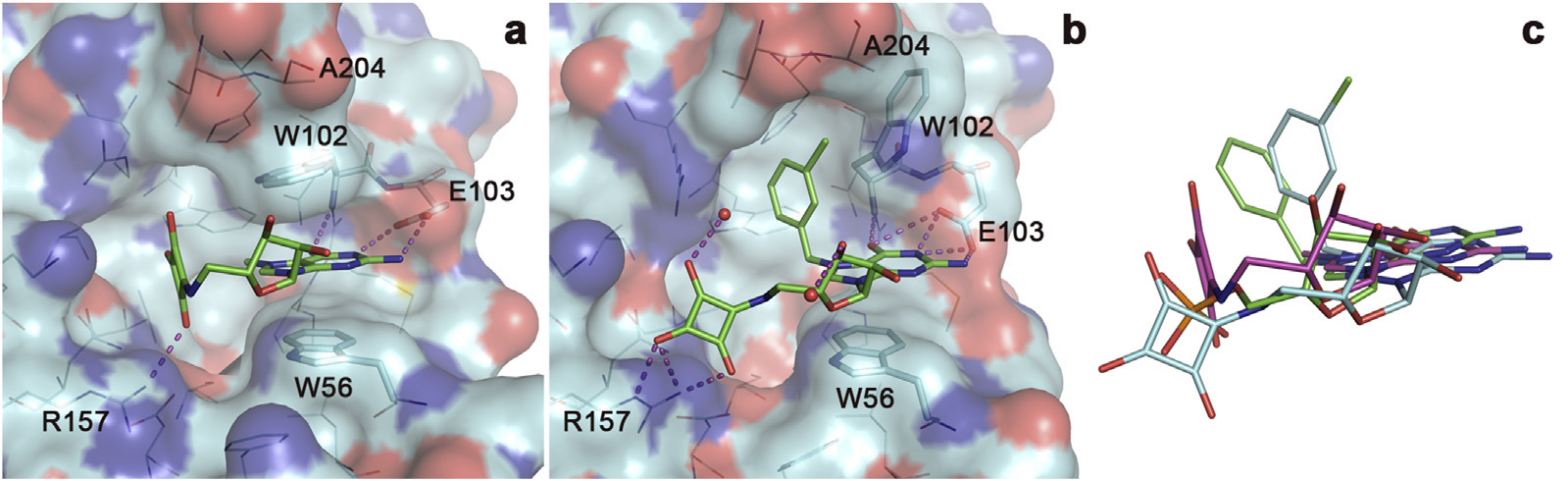
X-ray crystallographic eIF4E-binding poses of compound **4a** (**a**) and **4f** (**b**). (**a**) Alignment of experimental binding poses of Bn^7^GMP (PDB code 2V8X; green), **4a** (magenta), and **4f** (cyan). (For interpretation of the references to colour in this figure legend, the reader is referred to the web version of this article.)

The squaramide complex structures eIF4E:4EBP:**4a** and eIF4E:**4f** were solved to 3.61 Å and 2.40 Å, respectively (Table 3). Despite low overall resolution, the eIF4E co-crystal structure with ligand **4a** clearly shows that the squaramide moiety acts as a phosphate mimic by making a similar interaction with R157 despite an altered conformation (Fig. 7a). The positively charged N^7^-methyl guanine system forms the characteristic cation-π interaction as expected. Fig. 7b shows compound **4f** bound to the cap-binding site of eIF4E. The guanine group is located in its normal binding position, stacking with the side-chain of W56. As predicted, the squaramide occupies the α-phosphate position (Fig. 7c), making interactions with the side-chain of R157. The chloro substituent of the N^7^-benzyl group causes a rotation of approximately 90° in relation to other N^7^-benzyl substituted cap analogues (Fig. 7c). In turn, to accommodate the extra space required by the chloro group, the side chain of W102 is re-oriented away from making the usual cation-π stacking interaction to a position similar to that seen with **7a** in the presence of sulfate (compare Fig. 6, Fig. 7b).

**Fig. 8:**
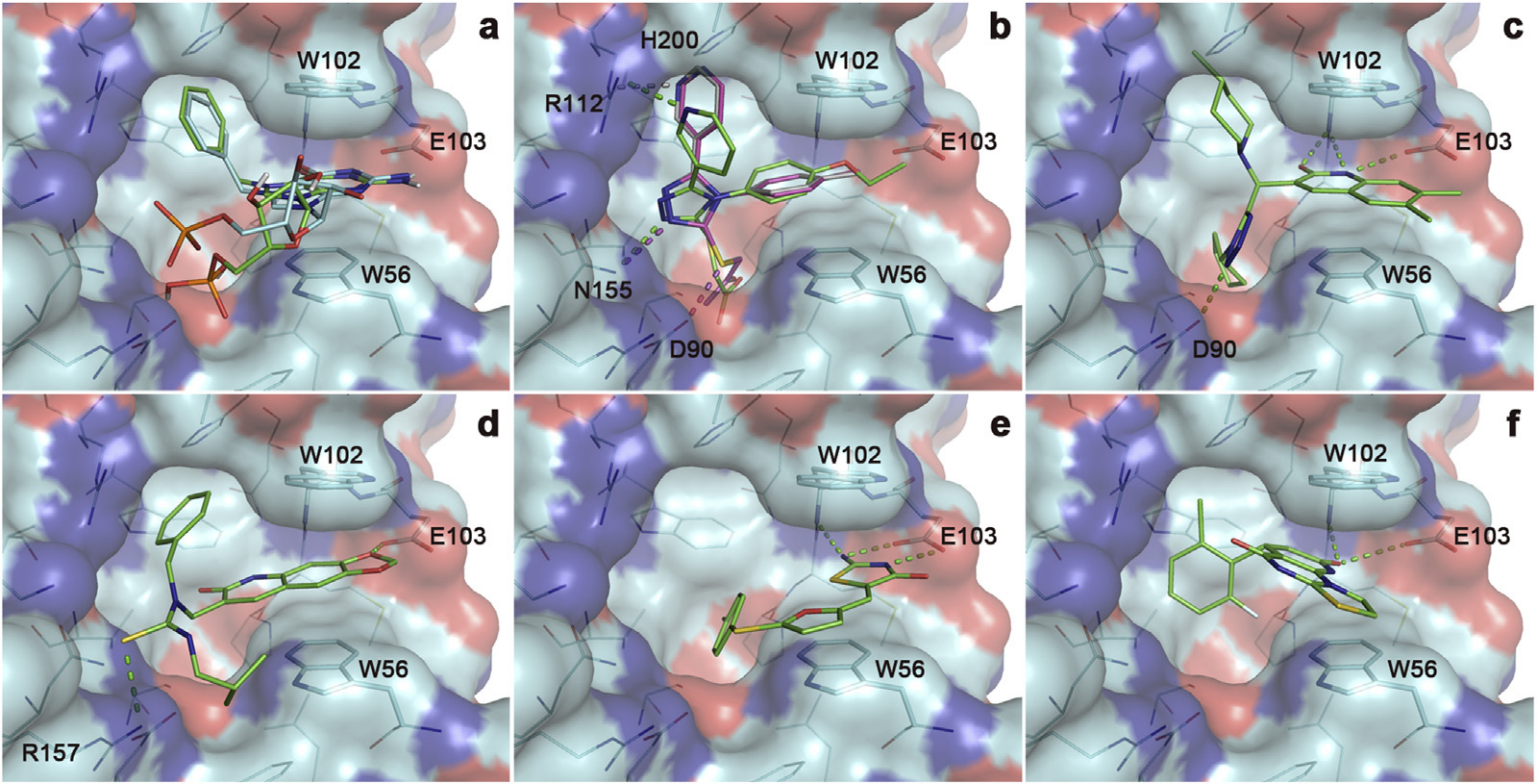
Predicted eIF4E binding poses of compounds **12**–**16**. (**a**) eIF4E binding pose of Bn^7^GMP as determined crystallographically (PDB code 2V8X; cyan), compared with re-docked pose of Bn^7^GMP (green) using the same docking protocol as in the virtual screen. Docked poses of virtual hits (**b**) **12a** (green), **12b** (grey), and **12c** (magenta); (**c**) **13**; (**d**) **14**; (**e**) **15**); (**f**) **16**. (For interpretation of the references to colour in this figure legend, the reader is referred to the web version of this article.)

We were unable to obtain complex crystal structures with unambiguous ligand density of the eIF4E inhibitors **12**–**16** from the virtual screen. However, it is interesting to compare the predicted binding modes of these compounds with the experimental eIF4E complexes of the m^7^GMP-derived derivatives (Fig. 8). Re-docking of Bn^7^GMP to the receptor structure of the Bn^7^GMP complex using the protocol used for virtual screening showed (Fig. 8a) virtually identical positioning of the N^7^-benzylguanine portion of the ligand, whereas the phosphate group in the re-docked pose bound more deeply into the cavity behind W56 and making polar interactions with the side chains of R157, W56, and D90, compared to the experimental binding pose, in which the phosphate group only interacts with R157. As a result of the different positioning of the phosphate group, the ribose systems in the experimental and modelled poses are also positioned somewhat differently.

The top-ranking docking solutions for the 4,5-bisaryl-2-carboxymethylthio-1,2,3-triazoles **12a**–**c** all showed similar poses, with the substituted phenyl groups stacking between W56 and W102, the unsubstituted aryl group inserting into the cavity behind W102, and the carboxymethyl substituents occupying the polar pocket behind W56 (Fig. 8b). Quinolinones **13** and **14** are predicted to stack between W56 and W102 in a similar manner, disposing the remaining substituents into the subpockets behind the W residues as shown (Fig. 8c and d). Thiazolone **15** and the pyridothiazolopyrimidine **16** again show stacked aromatic systems involving W56 and W102 but these ligands do not show good interactions with the other subpockets of the cap-binding site.

## 3 Conclusions

In the present study we report a new set of nucleotide-based cap analogues with phosphate modifications. Crystal structures of sulfonamide **7a** and squaramides **4a** and **4f** in complex with eIF4E were obtained and show similar binding modes to cap derivatives co-crystallised with eIF4E previously. However, our structures highlight the flexibility of the m^7^G-binding pocket, especially residue W102 that forms part of the cation-π interaction characteristic of cap-recognition by eIF4E. This finding suggests that cap-binding inhibitors of eIF4E devoid of a permanent cationic structure may be possible. All of the confirmed eIF4E inhibitors (**12a**, **14**, **16**) from our alternative design strategy based on virtual screening of lead-like non-nucleotide compounds support this suggestion. We present likely eIF4E-binding modes for these compounds but the possibility that these induce conformational changes in the cap-binding pocket of eIF4E cannot be excluded.

As has been found elsewhere [20], [25], imparting sufficient membrane permeability in eIF4E cap-binding inhibitors for such compounds effectively to block cellular eIF4E function is difficult. Despite modulation of the ionic and polar nature of the phosphate group in m^7^GMP derivatives through replacement with phosphate isosteres, none of our nucleoside monophosphate mimetic compounds showed cellular activity. The situation was different with the non-nucleotidic eIF4E inhibitors identified through virtual screening. These compounds possess physicochemical properties (Table 1) consistent with good permeability and we show that one of these compounds, the quinolinone **14**, not only inhibited eIF4E cap-binding in a biochemical and cell-free functional assay, but also acted as a protein synthesis inhibitor at the level of RNA translation in cellular assays. Although compound **14** is not particularly lead-like due to excessive ring count and lipophilicity, as well as low intrinsic solubility (Table 1), our results confirm for the first time that cellular eIF4E can be targeted with non-nucleotidic compounds and inherently drug-like compounds.

## 4 Materials and methods

### 4.1 Chemistry

Flash chromatography was carried out using silica gel cartridges (Sorbsil^®^ and ISOLUTE^®^). TLC was performed using Merck silica gel 60 F254 plates and visualisation by UV irradiation at 254 nm or by staining with KMnO_4_ or ninhydrin solutions, as appropriate. ^1^H- ^13^C-, ^19^F-, and ^31^P NMR spectra were obtained at room temperature using a Bruker 400 Ultrashield spectrophotometer at 400.13 MHz, 100.61 MHz, 376.50 MHz, and 161.97 MHz, respectively. Samples were prepared in the deuterated solvents Me_2_SO-*d*_6_ (unless otherwise indicated). Chemical shifts (*δ*) are expressed in ppm relative to SiMe_4_. Mass spectra were obtained using a Waters 2759 spectrometer using both positive and negative electrospray ionisation. Infrared spectra were recorded on a Nicolet IR-200 FT-IR spectrophotometer using the KBr disc technique. Analytical RP-HPLC was performed with a Shimadzu UFLCXR system coupled to a mass spectrometer (Applied Biosystems API2000). Phenomenex columns thermostated at 40 °C were used: C_8_-110A (method 1); Luna 3u (PFP2) 110A, (method 2), and C_18_ (method 3); all with dimensions 50 × 2 mm. The flow rate was 0.5 mL/min and UV detection was at 220 and 254 nm. Gradient elution: pre-equilibration for 1 min with 10% solvent B in solvent A, 10–98% solvent B over 2 min, 98% B for 2 min, 98 to 10% B over 0.5 min, and finally 10% B for 1 min (solvent A: 0.1% HCOOH in H_2_O, solvent B: 0.1% HCOOH in MeCN). Preparative RP-HPLC was carried out using a Waters automated LC system, with Waters 2525 pump, Waters 2767 sample manager, and Waters 2487 dual wavelength absorbance detector system or a Dionex ICS-3000 HPLC system with an Ultimate-3000 detector, using Phenomenex Luna PFP or HILIC (5 μM) columns (250 mm × 10 mm). The purity of all compounds screened in the biological assays was examined by LC−MS analysis and was found to be ≥ 95%.

### 4.2 *N*^2^-(4,4′-dimethoxytrityl)-2′,3′-*O*-(1-methylethylidene)guanosine (**1b**)

2′,3′-*O*-(1-Methyl-ethylidene)guanosine **1a** (mp 202–204 °C, lit [53] > 200 °C; 5 g, 15.5 mmol) was co-evaporated with pyridine (3 × 30 mL) and dissolved in dry pyridine (100 mL) under N_2_. Me_3_SiCl (9.8 mL, 77.4 mmol) was added over 5 min by syringe. The solution was stirred for 3 h, when DMTrtCl (6.3 g, 18.5 mmol) was added. Stirring (under N_2_) was continued for 21 h. The reaction was quenched with NH_4_OH (30 mL of a 35% solution in H_2_O). The mixture was stirred for 2 h and evaporated. The residue was dissolved in EtOAc (100 mL) and the solution was washed with H_2_O, brine, and aq NaHCO_3_ (50 mL each). The organic layer was evaporated and the residue was chromatographed (SiO_2_; CH_2_Cl_2_—MeOH, 9:1) to afford the title compound as a white solid (76%). Mp 179–181 °C. ^1^H NMR: *δ* 10.66 (bs,1H, NH), 7.82 (s, 1H), 7.58 (s, 1H) 7.17–7.29 (m, 9H), 6.84–6.86 (m, 4H), 5.38 (d, *J* = 3.9 Hz, 1H), 4.76 (dd, *J*_1_ = 6.2 Hz, *J*_2_ = 3.9 Hz, 1H), 4.39 (dd, *J*_1_ = 6.2 Hz, *J*_2_ = 2.4 Hz, 1H), 3.96 (td, *J*_1_ = 6 Hz, *J*_2_ = 2.4 Hz, 1H), 3.71 (s, 6H), 3.27–3.35 (m, 2H), 1.35 (s, 3H), 1.20 (s, 3H). ^13^C NMR: *δ* 157.67, 156.39, 150.94, 149.29, 145.10, 136.93, 136.85, 136.61, 129.72, 129.61, 128.24, 127.61, 126.47, 117.39, 112.92, 88.86, 85.14, 81.78, 80.94, 69.48, 61.39, 54.94, 27.09, 25.45. IR (KBr): *ν* 3342, 1685, 1607, 1568, 1508 cm^−1^. MS (ESI^+^): *m/z* [M+H]^+^ calcd for C_34_H_36_N_5_O_7_, 626.3; found: 626.1.

### 4.3 5′-Deoxy*-N*^2^-(4,4′-dimethoxytrityl)-5′-iodo-2′,3′-*O*-(1-methylethylidene)guanosine (**1c**)

A stirred solution of **1b** (1 g, 1.6 mmol) in anhydrous THF (100 mL) was cooled to −78 °C. (PhO)_3_P^+^MeI^−^ (1.45 g, 3.2 mmol) was added and the solution was allowed to reach r.t. over 30 min. THF was evaporated and the residue was diluted with EtOAc (50 mL). This solution was washed with Na_2_S_2_O_3_ (3 × 30 mL of a 25% solution in H_2_O). The organic phase was treated with MgSO_4_, filtered, and evaporated. The residue was dissolved in EtOAc (15 mL) and precipitated with hexane (100 mL). The precipitate was chromatographed (SiO_2_; CH_2_Cl_2_—MeOH, 9.2:0.8). The product was again precipitated from EtOAc–hexane to afford the title compound as a white solid (70%). Mp 148–150 °C. ^1^H NMR: *δ* 10.76 (s, 1H, NH), 7.81 (s, 1H), 7.57 (s, 1H) 7.11–7.35 (m, 9H), 6.87–6.89 (d, *J* = 10 Hz, 4H), 5.53 (d, *J* = 4.2 Hz, 1H), 4.66 (dd, *J*_1_ = 4.2 Hz, *J*_2_ = 6.2 Hz, 1H), 4.18 (dd, *J*_1_ = 6.2 Hz, *J*_2_ = 2.4 Hz, 1H), 4.04 (td, *J*_1_ = 6.2 Hz, *J*_2_ = 2.4 Hz, 1H), 3.75 (s, 6H), 2.77–2.91 (m, 2H), 1.40 (s, 3H), 1.21 (s, 3H). ^13^C NMR: *δ* 157.81, 156.34, 151.09, 149.06, 144.95, 137.49, 136.85, 136.64, 129.59, 129.51, 128.19, 127.76, 126.70, 118.00, 113.44, 113.05, 113.03, 89.49, 83.72, 82.78, 80.45, 69.41, 55.00, 27.09, 25.52, 5.75. IR (KBr): *ν* 3344, 1688, 1606, 1567, 1508 cm^−1^. MS (ESI^+^): *m/z* [M+H]^+^ calcd for C_34_H_35_IN_5_O_6,_ 736.16; found, 736.14.

### 4.4 5′-Azido*-*5′-deoxy*-N*^2^-(4,4′-dimethoxytrityl)-2′,3′-*O*-(1-methylethylidene)guanosine (**1d**)

To a stirred solution of **1c** (1.95 g, 2.65 mmol) in dry DMF (50 mL) NaN_3_ (490 mg, 7.5 mmol) was added under N_2_ and the reaction was heated to 85 °C for 24 h [CAUTION! Use of inorganic azide: reaction carried out behind a safety shield]. After cooling, the solution was evaporated and the residue was treated with H_2_O (40 mL). The mixture was stirred for 30 min. The precipitate was filtered and washed with H_2_O (2 × 30 mL) and Et_2_O (2 × 30 mL) and was then dried to afford the title compound as a white solid (77%). Mp 136–138 °C. ^1^H NMR: *δ* 10.76 (bs, 1H, NH), 7.81 (s, 1H), 7.57 (s, 1H), 7.13–7.35 (m, 9H), 6.86–6.90 (m, 4H), 5.53 (d, *J* = 3.9 Hz, 1H), 4.72 (dd, *J*_1_ = 6.2 Hz, *J*_2_ = 3.9 Hz, 1H), 4.17 (dd, *J*_1_ = 6.2 Hz, *J*_2_ = 2.4 Hz, 1H), 4.04 (m, 1H), 3.75 (s, 6H), 3.00–3.08 (m, 2H), 1.38 (s, 3H), 1.21 (s, 3H). ^13^C NMR: *δ* 157.80, 156.36, 151.12, 149.08, 144.97, 137.42, 136.84, 136.78, 129.58, 129.52, 128.20, 127.77, 126.65, 117.92, 113.34, 113.04, 89.04, 82.63, 81.14, 80.59, 69.40, 54.98, 51.36, 27.09, 25.53. IR (KBr): *ν* 3353, 2102, 1686, 1606, 1568, 1508 cm^−1^. MS (EMI^+^): *m/z* [M+H]^+^ calcd for C_34_H_35_N_8_O_6_, 651.27; found, 651.24.

### 4.5 5′-Amino*-*5′-deoxy*-N*^2^-(4,4′-dimethoxytrityl)-2′,3′-*O*-(1-methylethylidene)guanosine (**1e**)

To a stirred solution of **1d** (1 g, 1.5 mmol) in dry pyridine (15 mL) Ph_3_P (840 mg, 3.1 mmol) was added under N_2_ and the reaction was stirred for 3 h. The mixture was cooled to 0 °C and concentrated. NH_4_OH (2.8 mL of 35% solution in H_2_O) was added. Stirring was continued for 20 h, when the mixture was evaporated. The residue was chromatographed (Si_2_O; CHCl_3_—MeOH—Et_3_N, 9:1:0.1) to afford the title compound as a white solid (48%). Mp 203–205 °C. ^1^H NMR: *δ* 10.76 (bs, 1H, NH), 7.81 (s, 1H), 7.57 (s, 1H) 7.14–7.30 (m, 9H), 6.86 (dd, *J*_1_ = 8.8 Hz, *J*_2_ = 1.6 Hz, 4H), 5.45 (d, *J* = 3.9 Hz, 1H), 4.76 (dd, *J*_1_ = 6.4 Hz, *J*_2_ = 3.9 Hz, 1H), 4.23 (dd, *J*_1_ = 6.4 Hz, *J*_2_ = 3.0 Hz, 1H), 3.87–3.95 (m, 1H), 3.75 (s, 6H), 2.59–2.67 (m, 2H), 1.37 (s, 3H), 1.20 (s, 3H). ^13^C NMR: *δ* 157.71, 156.35, 151.15, 149.17, 144.99, 137.19, 137.02, 129.62, 128.23, 127.68, 126.55, 117.66, 113.40, 112.97, 88.51, 83.40, 81.17, 81.10, 69.46, 55.00, 42.30, 27.11, 25.62. IR (KBr): *ν* 3374, 1683, 1606, 1570, 1508 cm^−1^. MS (ESI^+^): *m/z* [M+H]^+^ calcd for C_34_H_37_N_6_O_6_, 625.28; found, 625.26.

### 4.6 5′-Deoxy*-N*^2^-(4,4′-dimethoxytrityl)-5′-(1,2-dioxo-3-methoxycyclobut-3-en-4-yl)amino-2′, 3′-*O*-(1-methylethylidene)guanosine (**2**)

To a stirred solution of **1e** (300 mg, 0.48 mmol) in MeOH (25 mL) iPr_2_NEt (40 μL, 1.92 mmol) was added. After 5 min, dimethyl squarate (68 mg, 0.48 mmol) was added to the reaction mixture, which was stirred for 4 h. It was then evaporated and the residue was chromatographed (Si_2_O; CHCl_3_—MeOH, 9:1) to afford the title compound as a white solid (61%). Mp 170–172 °C. ^1^H NMR: *δ* 10.77 (s, 1H), 8.5–8.7 (m, 1H), 7.74 (s, 1H), 7.60 (s, 1H) 7.11–7.33 (m, 9H), 6.88 (d, *J* = 8.4 Hz, 4H), 5.49 (dd, *J*_1_ = 14.4 Hz, *J*_2_ = 4 Hz, 1H), 4.61 (m, *J* = 3.6 Hz, 1H), 4.21–4.28 (m, 4H), 3.94 (m, *J* = 2.4 Hz, 1H), 3.68 (s, 6H), 2.80–3.26 (m, 1H), 1.38 (s, 3H), 1.21 (s, 3H). ^1^H NMR (75 °C): *δ* 10.61 (s, 1H), 8.41 (s, 1H), 7.68 (s, 1H), 7.42 (s, 1H), 7.16–7.31 (m, 9H), 6.83–6.88 (m, 4H), 5.46 (d, 1H, *J* = 3.6 Hz), 4.8 (dd, 1H, *J*_1_ = 6.3 Hz, *J*_2_ = 3.6 Hz), 4.33 (dd, 1H, *J*_1_ = 6.3 Hz, *J*_2_ = 3.1 Hz), 4.24 (s, 3H), 4.00 (dd, 1H, *J*_1_ = 6.3 Hz, *J*_2_ = 3.1 Hz), 3.72 (s, 6H), 3.03 (s, 2H), 1.38 (s, 3H), 1.21 (s, 3H). ^13^C NMR: *δ* 189.25, 189.04, 182.53, 177.62, 177.16, 172.29, 171.93, 157.78, 156.37, 151.04, 149.00, 145.01, 137.83, 136.95, 129.63, 129.50, 128.19, 127.82, 126.64, 118.07, 113.21, 113.06, 89.11, 88.95, 82.97, 82.58, 81.09, 80.93, 80.36, 80.19, 69.34, 60.10, 59.96, 54,97, 45.80, 45.14,27.15, 25.66. IR (KBr): *ν* 3412, 2935, 1702, 1608, 1508 cm^−1^. MS (ESI^+^): *m/z* [M+H]^+^ calcd for C_39_H_39_N_6_O_9_, 735.28; found, 735.24.

### 4.7 General method 1: N^7^-Alkylation of 5′-amino-5′-deoxy*-N*^2^-(4,4′-dimethoxytrityl)-2′,3′-*O*-(1-methylethylidene)guanosine derivatives **2**, **5**, and **8**

To a solution of compound **2**, **5a**, **5b**, or **8** (1 eq) in DMF, the appropriate alkyl or aryl halide (10 eq) was added and the mixture was stirred for 24–48 h, with monitoring by TLC (CHCl_3_—MeOH, 17:3) and LC-MS. After completion of reaction, the mixture was evaporated *in vacuo* and the residue was triturated with Et_2_O with the aid of sonication. The precipitate was filtered and chromatographed (SiO_2_; CHCl_3_—MeOH, 9:1) to afford the pure title compound.

### 4.8 5′-Deoxy*-N*^2^-(4,4′-dimethoxytrityl)-5′-(1,2-dioxo-3-methoxycyclobut-3-en-4-yl)amino-*N*^7^-methyl-2′,3′-*O*-(1-methylethylidene)guanosine (**3a**)

Using general method 1 from **2** and methyl bromide. White solid (57%). Mp 183–185 °C. ^1^H NMR: *δ* 9.88 (s, 1H), 8.78 (m, 1H), 8.29 (m, 1H), 7.66–7.88 (m, 10H), 7.27–7.34 (m, 4H), 6.1 (m, 1H), 5.53 (m, 1H), 5.1 (m, 1H), 4.82 (m, 4H), 4.58 (s, 3H), 4.19–4.22 (m, 2H), 4.20 (s, 6H), 1.89 (s, 3H), 1.64 (s, 3H). ^13^C NMR: *δ* 188.88, 188.58, 182,81, 177.31, 172.32, 171.82, 157.94, 157,85, 153.04, 147.17, 144.30, 138.09, 136.16, 129.69, 129.60, 128.22, 127.92, 126.90, 116.95, 113.16, 108.60, 91.65, 86.12, 85.77, 85.05, 80.94, 69.80, 60.27, 55.04, 45,64, 35.69, 26.74, 25.36. MS (ESI^+^): *m/z* [M]^+^ calcd for C_40_H_41_N_6_O_9_, 749.29; found, 749.26.

### 4.9 *N*^7^-Benzyl-5′-deoxy*-N*^2^-(4,4′-dimethoxytrityl)-5′-(1,2-dioxo-3-methoxycyclobut-3-en-4-yl)amino-2′,3′-*O*-(1-methylethylidene)guanosine (**3b**)

Using general method 1 from **2** and benzyl bromide. White solid (47%). Mp 182–185 °C. ^1^H NMR: *δ* 11.60 (s, 1H), 9.47 (s, 1H), 8.55–8.80 (m, 1H, NH), 8.14 (s, 1H), 7.14–7.42 (m, 14H), 6.82–6.85 (m, 4H), 5.6 (bs, 1H), 5.53 (m, 2H), 4.76–4.9 (m, 1H), 4.12–4.44 (m, 5H), 3.68 (s, 6H), 3.07 (m, 2H), 1.38 (s, 3H), 1.15 (s, 3H). ^13^C NMR: *δ* 189.29, 189.11, 182.69, 182.36, 177.91, 177.37, 172.26, 171.78, 157.80, 154.06, 147.70, 144.52, 137.37, 136.24, 134.10, 129.76, 129.67, 128.73, 128.68, 128.31, 127.79, 126.73, 116.91, 113.03, 107.80, 92.15, 86.32, 85.39, 81.04, 69.68, 60.25, 60.10, 54.97, 51.42, 45.40, 44.97, 26.79, 25.42. MS (ESI^+^): *m/z* [M]^+^ calcd for C_46_H_45_N_6_O_9,_ 825.32; found, 825.29.

### 4.10 5′-Deoxy*-N*^2^-(4,4′-dimethoxytrityl)-5′-(1,2-dioxo-3-methoxycyclobut-3-en-4-yl)amino-*N*^7^-(3-methylbenzyl)-2′,3′-*O*-(1-methylethylidene)guanosine (**3c**)

Using general method 1 from **2** and 3-methylbenzyl bromide. Off-white solid (67%). ^1^H NMR: *δ* 9.44 (s, 1H), 8.54–8.84 (m, 1H, NH), 7.10–7.33 (m, 14H), 6.79–6.88 (m, 4H), 5.64 (m, 1H), 5.31–5.48 (m, 2H), 4.79–4.94 (m, 1H), 4.12–4.41 (m, 5H), 3.66 (s, 6H), 2.93–3.28 (m, 2H), 2.26 (s, 3H), 1.38 (s, 3H), 1.15 (s, 3H). ^13^C NMR: *δ* 189.29, 189.08, 182.63, 182.32, 177.91, 177.33, 172.24, 171.77, 157.76, 154.96, 154.69, 147.72, 144.62, 137.95, 137.23, 136.40, 133.97, 129.80, 129.72, 129.41, 128.63, 127.75, 126.66, 125.83, 112.99, 107.74, 92.18, 86.29, 85.29, 81.73, 69.69, 60.52, 60.10, 54.96, 51.30, 45.29, 44.95, 26.78, 25.46, 20.94. MS (ESI^+^): *m/z* [M]^+^ calcd for C_47_H_47_N_6_O_9_, 839.3; found, 839.3.

### 4.11 5′-Deoxy*-N*^2^-(4,4′-dimethoxytrityl)-5′-(1,2-dioxo-3-methoxycyclobut-3-en-4-yl)amino-*N*^7^-(3-methoxybenzyl)-2′,3′-*O*-(1-methylethylidene)guanosine (**3d**)

Using general method 1 from **2** and 3-methoxybenzyl bromide. Off-white solid (54%). ^1^H NMR: *δ* 9.40 (s, 1H), 8.54–8.82 (m, 1H, NH), 6.79–7.32 (m, 18H), 5.6 (m, 1H), 5.39–5.49 (m, 2H), 4.72–4.94 (m, 1H), 4.09–4.31 (m, 5H), 3.72 (s, 3H), 3.67 (s, 6H), 2.90–3.25 (m, 2H), 1.38 (s, 3H), 1.15 (s, 3H). ^13^C NMR: *δ* 189.39, 189.05, 182.60, 182.28, 177.89, 177.38, 172.15, 171.85, 159.34, 157.72, 147.65, 144.70, 137.09, 136.50, 135.48, 129.90, 129.73, 128.30, 127.71, 126.62, 120.74, 118.43, 114.70, 113.9, 112.95, 107.85, 92.14, 86.73, 81.37, 79.16, 69.52, 60.19, 60.05, 55.08, 54.92, 51.16, 45.27, 44.97 26.74, 25.40. MS (ESI^+^): *m/z* [M]^+^ calcd for C_47_H_47_N_6_O_10_, 855.33; found, 855.32.

### 4.12 5′-Deoxy*-N*^2^-(4,4′-dimethoxytrityl)-5′-(1,2-dioxo-3-methoxycyclobut-3-en-4-yl)amino-2′, 3′-*O*-(1-methylethylidene)*-N*^7^-(3-(trifluoromethyl)benzyl)guanosine (**3e**)

Using general method 1 from **2** and 3-trifluoromethylbenzyl bromide. White solid (76%). ^1^H NMR: *δ* 9.43 (s, 1H), 8.52–8.82 (m, 1H, NH), 7.82–7.92 (m, 18H), 7.66–7.77 (m, 2H), 7.65–7.75 (m, 1H) 7.08–7.35 (m, 9H) 6.76–6.90 (m, 4H), 5.42–5.69 (m, 3H), 4.81–4.99 (m, 1H), 4.15–4.45 (m, 5H), 3.67 (s, 6H), 2.89–3.26 (m, 2H), 1.38 (s, 3H), 1.13 (s, 3H). ^13^C NMR: *δ* 189.32, 189.06, 182.61, 182.29, 178.75, 177.33, 172.30, 172.22, 157.71, 147.82, 144.75, 136.51, 135.42, 133.04, 129.81, 129.50, 128.73, 127.69, 126.59, 125.76, 125.56, 125.32, 124.82, 122.62, 118.12, 112.93, 107.75, 92.31, 86.66, 85.46 81.09, 69.57, 60.08, 54.93, 50.70, 44.92, 26.72, 25.40. ^19^F NMR (DMSO-*d*_6_): *δ* −60.98. IR (KBr): *ν* 3422, 2934, 1804, 1708, 1608, 1508 cm^−1^. MS (ESI^+^): *m/z* [M]^+^ calcd for C_47_H_44_F_9_N_6_O_9_, 893.3; found, 893.2.

### 4.13 *N*^7^-(3-Chlorobenzyl)-5′-deoxy*-N*^2^-(4,4′-dimethoxytrityl)-5′-(1,2-dioxo-3-methoxy-cyclo-but-3-en-4-yl)amino-2′,3′-*O*-(1-methylethylidene)guanosine (**3f**)

Using general method 1 from **2** and 3-chlorobenzyl bromide. Off-white solid (63%). Mp 202–204 °C. ^1^H NMR: *δ* 9.39 (s, 1H), 8.50–8.89 (m, 1H, NH), 7.10–7.62 (m, 14H), 6.74–6.93 (m, 4H), 5.63 (s, 1 H), 5.38–5.56 (m, 2H), 4.60–5.00 (m, 1H), 4.08–4.55 (m, 5H), 3.68 (s, 6H), 2.93–3.28 (m, 2H), 2.26 (s, 3H), 1.39 (s, 3H), 1.15 (s, 3H). ^13^C NMR: *δ* 189.32, 189.08, 182.64, 182.29, 177.94, 172.23, 171.81, 157.62, 147.87, 136.72, 136.45, 133.14, 130.49, 129.80, 129.75, 128.75, 128.46, 127.60, 126.46, 112.84, 107.65, 92.27, 90.43, 86,53 81.07, 69.48, 60.11, 54.92, 50.38, 45.65, 26.78, 25.46. IR (KBr): *ν* 3424, 2933, 1803, 1709, 1606, 1508 cm^−1^. MS (ESI^+^): *m/z* [M]^+^ calcd for C_46_H_44_ClN_6_O_9_, 859.3; found, 859.2.

### 4.14 5′-Deoxy*-N*^2^-(4,4′-dimethoxytrityl)-5′-(1,2-dioxo-3-methoxycyclobut-3-en-4-yl)amino-*N*^7^-(4-fluorobenzyl)-2′,3′-*O*-(1-methylethylidene)guanosine (**3g**)

Using general method 1 from **2** and 4-fluorobenzyl bromide. White solid (47%). Mp: 200–202 °C. ^1^H NMR: *δ* 11.87 (s, 1H), 9.37 (s, 1H), 8.44–8.85 (m, 1H, NH), 7.46–7.55 (m, 3H) 7.08–7.36 (m, 11H) 6.75–6.95 (m, 4H), 5.61 (s, 1H), 5.48 (m, 2H), 4.73–4.90 (m, 1H) 4.12–4.45 (m, 5H), 3.68 (s, 6H), 2.90–3.26 (m, 2H), 1.38 (s, 3H), 1.15 (s, 3H). ^13^C NMR: *δ* 189.47, 189.10, 182.58, 182.31, 178.49, 172.03, 161.01, 157.76, 154.56, 147.72, 144.90, 138.19, 137.38, 136.47, 131.14, 130.37, 129.73, 129.65, 128.29, 127.76,126.66, 115.66, 115.46, 113.00, 107.75, 92.13, 86.55, 81.01, 69.60, 60.10, 54.94, 50.81, 45.43, 26.76, 25.40. ^19^F NMR: *δ* −113.08. MS (ESI^+^): *m/z* [M]^+^ calcd for C_46_H_44_FN_6_O_9_, 843.3; found, 843.2.

### 4.15 5′-Deoxy*-N*^2^-(4,4′-dimethoxytrityl)-5′-(1,2-dioxo-3-methoxycyclobut-3-en-4-yl)amino-*N*^7^-(4-isopropylbenzyl)-2′,3′-*O*-(1-methylethylidene)guanosine **3h**

Using general method 1 from **2** and 4-isopropylbenzyl bromide. White solid (46%). Mp 184–186 °C. ^1^H NMR: *δ* 9.41 (s, 1H), 8.54–8.79 (m, 1H, NH), 7.11–7.38 (m, 14H), 6.75–6.91 (m, 4H), 5.63 (s, 1H), 5.32–5.52 (m, 2H), 4.73–4.91 (m, 1H), 4.09–4.41 (m, 5H), 3.67 (s, 6H), 2.86 (sp, *J* = 6.8 Hz, 1H), 2.26 (s, 3H), 1.38 (s, 3H), 1.1–1.0 (m, 10H). ^13^C NMR: *δ* 189.32, 189.13, 182.85, 177.96, 77.32, 172.24, 171.83, 157.78, 154.28, 149.09, 147.71, 147.70, 145.17, 136.62, 136.25, 131.57, 129.75, 128.82, 128.31, 127.78, 126.64, 113.01, 107.79, 92.15, 90.6, 86.25, 81.44, 81.08, 69.59, 59.75, 54.98, 51.12, 45.29, 33.16, 26.78, 25.43, 23.73. MS (ESI^+^): *m/z* [M]^+^ calcd for C_49_H_51_N_6_O_9_, 867.4; found, 867.3.

### 4.16 5′-Deoxy*-N*^2^-(4,4′-dimethoxytrityl)-5′-(1,2-dioxo-3-methoxycyclobut-3-en-4-yl)amino-*N*^7^-(3,5-(di-*tert*-butyl)benzyl)-2′,3′-*O*-(1-methylethylidene)guanosine (**3i**)

Using general method 1 from **2** to 3,5-(di-*tert*-butyl)benzyl bromide. White solid (48%). Mp 210–212 °C (dec). ^1^H NMR: *δ* 9.51 (s, 1H), 8.56–8.82 (m, 1H, NH), 8.33 (m, 1H), 7.11–7.45 (m, 13H), 6.75–6.90 (m, 4H), 5.58 (m, 1H), 5.37–5.49 (m, 2H), 4.76–4.92 (m, 1H), 4.02–4.52 (m, 5H), 3.66 (s, 6H), 2.87–3.27 (m, 2H), 1.38 (s, 3H), 1.24 (s, 18H), 1.10 (s, 3H). ^13^C NMR: *δ* 189.08, 182.59, 182.34, 177.92, 177.34, 172.24, 171.82, 157.73, 150.94, 147.74, 144.69, 136.39, 133.41, 129.84, 129.74, 128.40, 127.68, 126.63, 123.58, 122.58, 112.91, 107.91, 92.51, 86.82, 86.00, 81.65, 81.07, 69.67, 60.13, 54.91, 51.77, 45.23, 44.73, 34.55, 31.12, 26.70, 25.32. IR (KBr): *ν* 3434, 2958, 1802, 1708, 1607, 1508 cm^−1^. MS (ESI^+^): *m/z* [M]^+^ calcd for C_54_H_61_N_6_O_9_, 937.4; found, 937.4.

### 4.17 General method 2: deprotection of **3a**–**3i**

To a stirred solution of **3** (1 eq) in Me_2_CO, NaI (2 eq) was added and the mixture was heated under reflux for 48 h. After completion of the reaction, the solvent was evaporated and the residue was treated with HCOOH (1 mL/0.1 mmol of 80% aq soln) for 12 h. After cooling, the mixture was evaporated and the residue was triturated with Me_2_CO with the aid of sonication. The precipitate was filtered, washed successively with Me_2_CO and hexane, and was dried.

### 4.18 5′-Deoxy*-*5′-(1,2-dioxo-3-hydroxycyclobut-3-en-4-yl)amino-*N*^7^-methyl-guanosine (**4a**)

Using general method 2 from **3a**. White solid (38%). Mp 192–194 °C. ^1^H NMR: *δ* 9.48 (s, 1H), 8.11 (br s, 1H), 7.14–7.19 (m, 1H), 5.73 (d, *J* = 6.3 Hz, 1H), 5.51 (br s, 2H), 4.76 (t, *J* = 6.3 Hz, 1H), 4.13–4.22 (m, 2H), 4.02 (s, 3H), 3.98 (dd, *J*_1_ = 4.8 Hz, *J*_2_ = 2.6 Hz, 1H), 3.46–3.52 (m, 1H). ^13^C NMR: *δ* 197.95, 188.93, 180.62, 157.19, 154.06, 148.95, 137.79, 108.39, 90.36, 85.76, 71.59, 70.56, 44.67, 35.60. IR (KBr): *ν* 3367, 1794, 1709, 1674, 1619, 1600, 1549, 1519 cm^−1^. Anal. RP-HPLC: *t*_R_ 0.77 min (method 1, >95%). MS (ESI^+^): *m/z* [M]^+^ calcd for C_15_H_17_N_6_O_7,_ 393.12; found, 393.11.

### 4.19 *N*^7^-Benzyl-5′-deoxy*-*5′-(1,2-dioxo-3-hydroxy-cyclobut-3-en-4-yl)amino-guanosine (**4b**)

Using general method 2 from **3b**. White solid (35%). Mp 186–188 °C. ^1^H NMR: *δ* 11.70 (s, 1H), 9.75 (s, 1H), 7.52–7.57 (m, 2H), 7.36–7.43 (m, 3H), 7.11–7.18 (m, 1H), 5.76 (d, *J* = 6.0 Hz, 1H), 5.63 (s, 1H), 4.74 (d, *J*_1_ = 10.8 Hz, *J*_2_ = 5.8 Hz, 1H), 4.12–4.26 (m, 2H), 3.98 (dd, *J*_1_ = 10.8 Hz, *J*_2_ = 4.2 Hz, 1H), 3.48–3.53 (m, 1H). ^13^C NMR: *δ* 197.88, 189.00, 180.63, 156.74, 154.46, 149.28, 137.58, 134.48, 128.76, 128.63, 128.54, 107.58, 90.65, 85.66, 71.73, 70.60, 51.43, 44.72. IR (KBr): *ν* 3394, 1791, 1706, 1632, 1606, 1532 cm^−1^. Anal. RP-HPLC: *t*_R_ 2.33 min (method 1, >95%), *t*_R_ 1.51 min (method 2, >94%). MS (ESI^+^): *m/*z [M]^+^ calcd for C_21_H_21_N_6_O_7_, 469.15; found, 469.14.

### 4.20 5′-Deoxy*-*5′-(1,2-dioxo-3-hydroxycyclobut-3-en-4-yl)amino-*N*^7^-3-methylbenzyl-guano-sine (**4c**)

Using general method 2 from **3c**. White solid (37%). Mp 187–190 °C. ^1^H NMR: *δ* 11.75 (br s, 1H), 9.80 (s, 1H), 7.28–7.35 (m, 2H), 7.15–7.22 (m, 1H) 7.06–7.15 (m, 1H), 5.77 (d, *J* = 5.9 Hz, 1H), 5.57 (s, 2H), 4.73 (t, *J* = 5.9 Hz, 1H), 4.13–4.22 (m, 4H), 3.98 (m, 1H), 3.47–3.58 (m, 1H), 2.30 (s, 3H). ^13^C NMR: *δ* 197.70, 188.90, 180.55, 155.79, 153.21, 149.17, 138.21, 137.96, 134.22, 129.44, 129.06, 128.84, 125.62, 107.59, 90.71, 85.61, 71.79, 70.58, 51.49, 44.74, 20.97. Anal. RP-HPLC: *t*_R_: 2.52 (method 1, >95%), *t*_R_: 1.98 min (method 2, >95%). MS (ESI^+^): *m/z* [M]^+^ calcd for C_22_H_23_N_6_O_7_, 483.16; found, 483.15.

### 4.21 5′-Deoxy*-*5′-(1,2-dioxo-3-hydroxycyclobut-3-en-4-yl)amino-*N*^7^-3-methoxybenzyl-guano-sine (**4d**)

Using general method 2 from **3d**. White solid (36%). Mp 177–179 °C. ^1^H NMR: *δ* 11.69 (s, 1H), 9.78 (s, 1H), 7.32 (m, 1H), 7.07–7.14 (m, 3H), 6.95 (m, 1H), 5.77 (d, *J* = 6.0 Hz, 1H), 5.58 (s, 2H), 5.51 (br s, 2H), 4.73 (t, *J* = 6.0 Hz, 1H), 4.11–4.21 (m, 2H), 3.98 (m 1H), 3.75 (s, 3H), 3.49–3.55 (m, 1H). ^13^C NMR: *δ* 197.50, 188.86, 180.38, 159.49, 155.74, 153.17, 149.17, 138.03, 135.64, 130.13, 120.60, 114.34, 114.18, 107.59, 90.71, 85.64, 71.75, 70.57, 55.17, 51.45, 44.74. Anal. RP-HPLC: *t*_R_ 2.42 min (method 1, >95%), *t*_R_ 1.92 min (method 2, >95%). MS (ESI^+^): *m/z* [M]^+^ calcd for C_22_H_23_N_6_O_8_, 499.16; found, 499.15.

### 4.22 5′-Deoxy*-*5′-(1,2-dioxo-3-hydroxycyclobut-3-en-4-yl)amino-*N*^7^-3-(trifluoromethyl)benz-yl-guanosine (**4e**)

Using general method 2 from **3e**. The crude product was purified by preparative HPLC using a Luna HILIC column *t*_R_ 8.0 min; gradient from 0% A to 100% A over 12.5 min, where A is 5:4:1 MeCN—H_2_O—50 mM aq NH_4_HCOO pH 3.2, and B is 9:1 MeCN–50 mM aq NH_4_HCOO pH 3.2, to afford 4e as a white solid (14.2%). Mp 221–224 °C. ^1^H NMR: *δ* 8.21 (s, 1H), 7.97 (s, 1H) 7.49–7.59 (m, 4H), 5.60–5.78 (m, 3H) 4.71 (dd, *J*_1_ = 10.4 Hz, *J*_2_ = 4.8 Hz, 1H), 4.08–4.27 (m, 2H), 3.98 (dd, *J*_1_ = 4.7 Hz, *J*_2_ = 3.2 Hz, 1H), 3.48–3.55 (m, 1H). ^13^C NMR: *δ* 197.79, 189.28, 181.22, 155.78, 153.24, 149.24, 138.20, 138.08, 135.47, 132.91, 130.07, 129.27, 125.60, 125.38, 107.58, 90.76, 85.54, 71.87, 70.52, 50.93, 44.69. ^19^F NMR: *δ* −61.02. Anal. RP-HPLC: *t*_R_: 2.67 min (method 1, >95%), *t*_R_ 2.15 min (method 2, >95%). MS (ESI^+^): *m/z* [M]^+^ calcd for C_22_H_20_F_3_N_6_O_7_, 537.13; found, 537.13.

### 4.23 *N*^7^-3-Chlorobenzyl-5′-deoxy*-*5′-(1,2-dioxo-3-hydroxycyclobut-3-en-4-yl)aminoguanosine (**4f**)

Using general method 2 from **3f**. White solid (44%). Mp 193–195 °C. ^1^H NMR: *δ* 11.73 (s, 1H), 9.80 (s, 1H), 7.42–7.66 (m, 4H), 7.13–7.18 (m, 1H) 5.77 (d, *J* = 6.0 Hz, 1H), 5.61 (s, 2H), 4.71 (t, *J* = 5.7 Hz, 1H), 4.11–4.21 (m, 2H), 3.98 (m, 1H), 3.52 (s, 3H), 3.51–3.56 (m, 1H). ^13^C NMR: *δ* 197.30, 188.80, 180.34, 155.76, 153.16, 149.25, 138.17, 136.54, 133.34, 130.83, 128.78, 128.45, 127.37, 107.55, 90.74, 85.49, 71.94, 70.55, 50.85, 44.76. Anal. RP-HPLC: *t*_R_ 2.54 min (method 1, >95%), *t*_R_ 2.02 min (method 2, >94%). MS (ESI^+^): *m/z* [M]^+^ calcd for C_21_H_20_ClN_6_O_7_, 503.11; found, 503.10.

### 4.24 5′-Deoxy*-*5′-(1,2-dioxo-3-hydroxycyclobut-3-en-4-yl)amino-*N*^7^-4-flurobenzyl-guanosine (**4g**)

Using general method 2 from **3g**. White solid (31%). ^1^H NMR: *δ* 9.69 (s, 1H), 7.64–7.70 (m, 2H), 7.23–7.26 (m, 4H), 5.74 (d, *J* = 5.9 Hz, 1H), 5.62 (s, 2H), 4.74 (dd, *J*_1_ = 5.9 Hz, *J*_2_ = 4.8 Hz, 1H), 4.12–4.25 (m, 2H), 3.98 (dd, *J*_1_ = 7.1 Hz, *J*_2_ = 3.2 Hz, 1H), 3.75 (s, 3H), 3.46–3.52 (m, 1H). ^13^C NMR: *δ* 197.94, 188.97, 180.73, 158.03, 156.09, 149.33, 136.87, 131.22, 131.14, 130.85, 115.83, 115.62, 107.50, 90.54, 85.54, 71.74, 70.61, 50.53, 44.76. ^19^F NMR: *δ* −113.16. Anal. RP-HPLC: *t*_R_ 2.44 min (method 1, >95%), *t*_R_ 1.90 min (method 2, >95%). MS (ESI^+^): *m/z* [M]^+^ calcd for C_21_H_20_FN_6_O_7_, 487.14; found, 487.14.

### 4.25 5′-Deoxy*-*5′-(1,2-dioxo-3-hydroxycyclobut-3-en-4-yl)amino-*N*^7^-4-isopropylbenzyl-guanosine (**4h**)

Using general method 2 from **3h**. White solid (11%). Mp 167–169 °C. ^1^H NMR: *δ* 9.64 (s, 1H), 8.23 (s, 1H), 7.48 (d, *J* = 8.0 Hz, 2H), 7.27 (d, *J* = 8.0 Hz, 3H), 5.73 (d, *J* = 6.3 Hz, 1H), 5.30–5.60 (m, 4H), 4.79 (t, *J* = 6.3 Hz, 1H), 4.09–4.30 (m, 2H), 3.96 (m, 1H), 3.45–3.51 (m, 1H), 2.87 (sp, *J* = 6.8 Hz, 1H), 1.17 (d, *J* = 6.8 Hz, 6H). ^13^C NMR: *δ* 197.78, 188.98, 180.52, 155.77, 153.16, 149.10, 147.89, 137.85, 135.17, 128.69, 127.50, 126.83, 126.48, 107.50, 90.71, 85.62, 71.79, 70.55, 51.27, 44.68, 33.17, 33.09, 23.79, 23.73. Anal. RP-HPLC: *t*_R_: 2.73 min (method 1, >95%), *t*_R_ 2.21 min (method 2, >95%). MS (ESI^+^): *m/z* [M]^+^ calcd for C_24_H_27_N_6_O_7_, 511.19; found, 511.19.

### 4.26 *N*^7^-3,5-(di-*tert*-butyl)benzyl-5′-deoxy*-*5′-(1,2-dioxo-3-hydroxycyclobut-3-en-4-yl)amino-guanosine (**4i**)

Using general method 2 from **3i**. White solid (15%). ^1^H NMR: *δ* 9.53 (s, 1H), 8.40 (s, 2H), 8.28 (s, 1H), 7.45 (d, *J* = 1.65 Hz, 2H), 7.37 (m, 1H), 5.73 (d, *J* = 6.9 Hz, 1H), 5.62 (s, 2H), 4.89 (dd, *J*_1_ = 6.9 Hz, *J*_2_ = 4.8 Hz, 1H), 4.21 (td, *J*_1_ = 8.1 Hz, *J*_2_ = 3.2 Hz, 1H), 4.14 (dd, *J*_1_ = 4.8 Hz, *J*_2_ = 3.2 Hz, 1H), 3.96 (dd, *J*_1_ = 4.8 Hz, *J*_2_ = 1.8 Hz, 1H), 3.40 (m, 1H), 1.26 (s, 18H). ^13^C NMR: *δ* 198.14, 188.89, 180.75, 165.84, 150.93, 150.61, 149.23, 135.27, 134.31, 123.01, 122.20, 107.92, 90.46, 86.06, 70.93, 70.83, 51.30, 44.88, 31.19. Anal. RP-HPLC: *t*_R_ 3.07 min (method 1, >86%), *t*_R_ 2.54 min (method 2, >95%). MS (ESI^+^): *m/z* [M]^+^ calcd for C_29_H_37_N_6_O_7_, 581.27; found, 581.27.

### 4.27 5′-Deoxy*-N*^2^-(4,4′-dimethoxytrityl)-2′,3′-*O*-(1-methylethylidene)-5′-(trifluoromethyl-sulfamoyl)guanosine (**5a**)

To a stirred solution of **1e** (200 mg, 0.32 mmol) in dry CH_2_Cl_2_ (10 mL), Et_3_N (128 μL, 1.28 mmol) was added. The mixture was cooled to 0 °C and CF_3_SO_2_Cl (33 μL, 0.32 mmol) was added drop-wise *via* syringe. After stirring for 48 h, the mixture was evaporated and the residue was chromatographed (Si_2_O; CHCl_3_—MeOH, 9:1) to afford the title compound as a yellow oil (49%). ^1^H NMR: *δ* 10.78 (s, 1H), 9.70 (s, 1H), 7.82 (s, 1H), 7.63 (s, 1H), 7.13–7.34 (m, 9H), 6.88 (d, *J* = 7.5 Hz, 4H), 5.54 (d, *J* = 4.0 Hz, 1H), 4.72 (dd, *J*_1_ = 6.2 Hz, *J*_2_ = 4.0 Hz, 1H), 4.19 (dd, *J*_1_ = 6.2 Hz, *J*_2_ = 3.1 Hz, 1H), 3.87–3.93 (m, 1H), 3.72 (s, 6H), 2.93–3.09 (m, 2H), 1.39 (s, 3H), 1.21 (s, 3H). ^13^C NMR: *δ* 157.87, 156.47, 151.20, 149.22, 145.06, 137.49, 137.08, 136.89, 129.65, 129.60, 128.30, 127.78, 126.66, (q, *J* = 324 Hz, 124 Hz, 121 Hz, 118 Hz, 115 Hz, CF_3_), 124.35, 121.15, 117.94, 114.74, 117.89, 113.83, 113.08, 88.58, 82.91, 80.83, 80.81, 69.52, 54.98, 45.11, 27.16, 25.71. ^19^F NMR: *δ* −77.46. MS (ESI^+^): *m/z* [M+H]^+^ calcd for C_35_H_36_F_3_N_6_O_8_S, 757.2; found, 757.2.

### 4.28 5′-(4-Carboxyphenyl)sulfamoyl-5′-deoxy*-N*^2^-(4,4′-dimethoxytrityl)-2′,3′-*O*-(1-methyl-ethylidene)guanosine (**5b**)

To a stirred solution of **6e** (300 mg, 0.48 mmol) in CH_2_Cl_2_ (25 mL) Et_3_N was added (254 μL, 1.92 mmol). After cooling to 0 °C, 4-carboxybenzenesulfonyl chloride (105 mg, 0.48 mmol) was added and the mixture was allowed to reach r.t. and was stirred for 24 h. It was evaporated and the residue was chromatographed (Si_2_O; CH_2_Cl_2_—MeOH, 8.5:1.5) to afford the title compound as a white solid (73%). ^1^H NMR: *δ* 10.92 (bs, 1 H), 8.08 (dd, *J*_1_ = 6.8 Hz, *J*_2_ = 1.7 Hz, 2 H), 7.79 (dd, *J*_1_ = 6.8 Hz, *J*_2_ = 1.7 Hz, 2 H), 7.77 (s, 1 H), 7.76 (s, 1 H) 7.08–7.24 (m, 9 H), 6.78–6.92 (m, 4H), 5.39 (d, *J* = 4.1 Hz, 1 H), 4.74 (dd, *J*_1_ = 6.4 Hz, *J*_2_ = 4.1 Hz, 1H), 4.14 (dd, *J*_1_ = 6.4 Hz, *J*_2_ = 3.1 Hz, 1 H), 3.83 (td, *J*_1_ = 6.4 Hz, *J*_2_ = 3.1 Hz, 1 H), 3.68 (s, 6H), 2.67–2.74 (m, 2 H), 1.33 (s, 3 H), 1.17 (s, 3 H). ^13^C NMR: *δ* 173.09, 157.66, 151.19, 149.11, 144.97, 140.37, 137.16, 136.26, 129.85, 129.53, 128.17, 127.60, 126.25, 124.71, 117.71, 113.59, 112.92, 88.39, 82.69, 80.97, 69.44, 59.82, 44.42, 27.87, 25.58. IR (KBr): *ν* 3314, 2985, 2674, 2488, 1685, 1605, 1570, 1508 cm^−1^. MS (ESI^+^): *m/z* [M+H]^+^ calcd for C_41_H_41_N_6_O_10_ S 809.26 found: 809.23.

### 4.29 *N*^7^-Benzyl-5′-deoxy*-N*^2^-(4,4′-dimethoxytrityl)-2′,3′-*O*-(1-methylethylidene)-5′-(trifluoro-methylsulfamoyl)guanosine (**6a**)

Using general method 1 from **5a** and benzyl bromide. Light yellow solid (53%). ^1^H NMR: *δ* 11.42 (br s, 1H), 10.11 (s, 1H), 8.18 (s, 1 H), 7.45–7.53 (m, 2H), 7.15–7.41 (m, 13H), 6.74–6.93 (m, 4H), 5.34–5.51 (m, 3H), 4.99 (dd, *J*_1_ = 5.8 Hz, *J*_2_ = 1.3 Hz, 1H), 4.65 (d, *J* = 5.7 Hz, 1H), 4.30–4.39 (m, 1H), 3.70 (s, 6H), 3.03–3.10 (m, 2H), 1.37 (s, 3H), 1.11 (s, 3H). ^13^C NMR: *δ* 157.71, 152.92, 147.71, 144.44, 137.76, 136.24, 136.08, 134.33, 129.89, 129.73, 128.88, 128.62, 128.35, 127.60, 126.58, 112.90, 112.84, 111.86, 106.78, 92.42, 89.39, 83.96, 81.86, 69.69, 54.90, 51.53, 47.55, 43.65, 26.66, 24.97. ^19^F NMR: *δ* −76.09. IR (KBr): *ν* 3430, 2933, 1706, 1607, 1509. MS (ESI^+^): *m/z* [M]^+^ calcd for C_42_H_42_F_3_N_6_O_8_S, 847.27; found, 847.25.

### 4.30 5′-(4-Carboxyphenyl)sulfamoyl-5′-deoxy*-N*^2^-(4,4′-dimethoxytrityl)-*N*^7^-methyl-2′,3′-*O*-(1-methylethylidene)guanosine (**6b**)

Using general method 1 from **5b** and methyl iodide. The crude product was purified by SiO_2_ column chromatography (CHCl_3_—MeOH, 7.5:2) to furnish pure title compound as a white solid (61%). ^1^H NMR (MeOH-*d*_4_): *δ* 8.13 (d, *J* = 8.4 Hz, 2H) 7.85 (d, *J* = 8.4 Hz, 2 H), 7.16–7.38 (m, 9 H), 6.77–6.87 (m, 4H), 5.60 (d, *J* = 1.2 Hz,1 H), 5.09 (dd, *J*_1_ = 6 Hz, *J*_2_ = 2 Hz, 1 H), 4.33 (dd, *J*_1_ = 6 Hz, *J*_2_ = 1.7 Hz, 1 H), 4.26 (td, *J*_1_ = 6 Hz, *J*_2_ = 1.7 Hz, 1 H), 4.07 (s, 3 H) 3.74 (s, 6 H), 2.76–2.90 (m, 2H), 1.41 (s, 3 H), 1.11 (s, 3 H). ^13^C NMR (MeOH-*d*_4_): *δ* 160.08, 153.04, 149.21, 143.94, 139.37, 137.58, 131.27, 131.22, 129.88, 129.33, 128.99, 128.18, 127.78, 114.87, 114.23, 113.99, 113.02, 95.08, 88.21, 84.35, 83.41, 72.10, 65.31, 55.74, 36.65, 27.13, 25.82. MS (ESI^+^): *m/z* [M+2H]^+^ calcd for C_42_H_45_N_6_O_10_S, 823.3; found, 823.2.

### 4.31 *N*^7^-Benzyl-5′-(4-carboxyphenyl)sulfamoyl-5′-deoxy*-N*^2^-(4,4′-dimethoxytrityl)-2′,3′-*O*-(1-methylethylidene)guanosine (**6c**)

Using general method 1 from **5c** and benzyl bromide. The crude product was purified by SiO_2_ column chromatography (CHCl_3_—MeOH, 8:2) to furnish pure title compound as a white solid (58%). ^1^H NMR (MeOH-*d*_4_): *δ* 9.16 (s,1H), 8.14 (d, *J* = 8.5 Hz, 2 H), 7.80 (d, *J* = 8.5 Hz, 2H), 7.49–7.54 (m, 2 H), 7.35–7.39 (m, 3 H), 7.16–7.36 (m, 9 H), 6.74–6.83 (m, 4H), 5.54–5.68 (m, 3H), 5.04 (dd, *J*_1_ = 6.1 Hz, *J*_2_ = 1.7 Hz, 1 H), 4.27 (dd, *J*_1_ = 6.1 Hz, *J*_2_ = 2.0 Hz, 1H), 4.17–4.23 (m, 1 H), 3.73 (s, 6 H), 2.75–2.86 (m, 2H), 1.39 (s, 3H), 1.10 (s, 3H). ^13^C NMR (MeOH-*d*_4_): *δ* 160.08, 154.91, 149.52, 145.95, 143.85, 137.61, 135.27, 131.22, 131.19, 129.87, 129.51, 129.01, 128.20, 127.78, 114.98, 114.25, 114.17, 109.76, 95.04, 88.06, 84.17, 82.94, 72.10, 55.73, 53.74, 43.81, 27.14, 25.87. MS (ESI^+^): *m/z* [M]^+^ calcd for C_48_H_47_N_6_O_10_S, 899.3; found, 899.2.

### 4.32 General method 3: deprotection of **6a**–**6c** and **9a**–**9b**

A solution of **6** or **9** in HCOOH (1 mL/0.1 mmol of 80% aq solution) was stirred for 12 h. The mixture was evaporated and the residue was triturated with Me_2_CO with the aid of sonication. The precipitate was filtered, washed successively with Me_2_CO and hexane, and was dried.

### 4.33 *N*^7^-Benzyl-5′-deoxy*-*5′-(trifluoromethylsulfamoyl)guanosine (**7a**)

Using general method 3 from **6a**. The crude product was purified by preparative HPLC using a Luna PFP column (*t*_R_ 4.9 min; isocratic elution at 35% B in A, where A is 0.1% CF_3_COOH in H_2_O, and B is 0.1% CF_3_COOH in MeCN) to afford 7a as a white solid (26%). ^1^H NMR: *δ* 9.27 (s, 1H), 7.49–7.56 (m, 2H), 7.36–7.44 (m, 3H), 5.94 (d, *J* = 4.0 Hz, 1H), 5.66 (dd, *J*_1_ = 21.0 Hz, *J*_2_ = 14.4 Hz, 2H), 4.19 (dd, *J*_1_ = 5.2 Hz, *J*_2_ = 4.0 Hz, 1H), 4.29 (t, *J* = 5.2 Hz, 1H), 4.13–4.17 (m, 1H), 3.57–3.63 (m, 2H). ^13^C NMR: *δ* 157.48, 154.98, 151.33, 134.99, 130.38, 130.27, 129.81, 122.94, 119.75, 109.10, 92.73, 85.28, 74.84, 72.12, 53.63, 46.45. ^19^F NMR: *δ* −76.39. Anal. RP-HPLC: *t*_R_ 2.63 min (method 1, >95%), *t*_R_ 2.23 min (method 2, >63%). MS (ESI^+^): *m/z* [M]^+^ calcd for C_18_H_20_F_3_N_6_O_6_S, 505.11; found, 505.11.

### 4.34 5′-(4-Carboxyphenyl)sulfamoyl-5′-deoxy*-N*^7^-methylguanosine (**7b**)

Using general method 3 from **6b**. The crude product was purified by preparative HPLC using a LUNA PFP column (*t*_R_ 4.0 min, gradient of 5%–50% B in A over 15 min, where A is 0.1% CF_3_COOH in H_2_O, and B is 0.1% CF_3_COOH in MeCN) to afford 7b as a white solid (44%). ^1^H NMR (DMSO-*d*_6_ and D_2_O): *δ* 9.26 (s, 1H), 8.28 (s, 1H), 8.11 (s, 1H), 8.00 (d, *J* = 7.7 Hz, 2H), 7.76 (d, *J* = 7.7 Hz, 2H), 7.58 (br s, 2H), 5.75 (d, *J* = 4.8 Hz, 1H), 4.50 (t, *J* = 4.8 Hz, 1H), 4.06 (t, *J* = 4.8 Hz, 1H), 4.01 (s, 3H), 3.97 (dd, *J*_1_ = 9.6 Hz, *J*_2_ = 4.8 Hz), 3.10 (m, 2H, H5′). ^13^C NMR (DMSO-*d*_6_ and D_2_O): *δ* 173.36, 171.03, 149.41, 140.95, 140.23, 129.31, 127.48, 126.57, 125.85, 109.03, 90.32, 83.60, 72.82, 70.51, 43.70, 35.74. Anal. RP-HPLC: *t*_R_ 1.09 min (method 1, >95%), *t*_R_ 0.77 min (method 2, >95%). MS (ESI^+^): *m/z* [M]^+^ calcd for C_41_H_40_N_6_O_10_S, 481.11; found, 481.11.

### 4.35 *N*^7^-Benzyl-5′-(4-carboxyphenyl)sulfamoyl-5′-deoxyguanosine (**7c**)

Using general method 3 from **6c**. The crude product was purified by RP-HPLC with a LUNA PFP column (*t*_R_ 19.8 min, gradient of 10%–30% B in A over 20 min, where A is 0.1% CF_3_COOH in H_2_O, and B is 0.1% CF_3_COOH in MeCN) to afford 7c as a white solid (38%). ^1^H NMR: *δ* 9.56 (s, 1H), 8.11 (s, 1H), 8.06 (d, *J* = 8.4 Hz, 2H), 7.87 (d, *J* = 8.4 Hz, 2H), 7.37–7.51 (m, 7H), 5.79 (d, *J* = 4.4 Hz, 1H), 5.59 (s, 2H), 4.50 (t, *J* = 4.8 Hz, 1H), 4.09 (t, *J* = 4.8 Hz, 1H), 3.89–4.02 (m, 1H), 3.09–3.24 (m, 2H). ^13^C NMR: *δ* 166.17, 155.77, 153.16, 149.62, 144.32, 136.48, 134.30, 130.00, 128.84, 128.20, 126.70, 106.79, 89.14, 83.71, 73.45, 70.58, 51.61, 49.29. Anal. RP-HPLC: *t*_R_ 2.49 min (method 1, >95%), *t*_R_ 2.09 min (method 2, >95%). MS (ESI^+^): *m/z* [M]^+^ calcd for C_24_H_25_N_6_O_8_S, 577.14; found, 577.14.

### 4.36 5′-(*tert*-Butyloxycarbamoyl)-5′-deoxy*-N*^2^-(4,4′-dimethoxytrityl)-2′,3′-*O*-(1-methylethyli-dene)guanosine (**8**)

To a stirred solution of **1e** (0.5 g, 0.8 mmol) in CH_2_Cl_2_ (30 mL) was added Et_3_N (220 μL, 1.6 mmol). The mixture was cooled to 0 °C and Boc_2_O (170 mg, 0.8 mmol) was added. Stirring was continued for 3 h, when the mixture was evaporated. The residue was purified by SiO_2_ column chromatography (CH_2_Cl_2_—MeOH, 9:1) to afford pure title compound as a white solid (375 mg, 65%). ^1^H NMR: *δ* 10.66 (s, 1H, NH), 7.76 (s, 1H), 7.58 (s, 1H) 7.10–7.33 (m, 9H), 6.83–6.89 (d, *J* = 8.5 Hz, 4H), 5.36 (d, *J* = 3.9 Hz, 1H), 4.76 (dd, *J*_1_ = 6.0 Hz, *J*_2_ = 3.9 Hz, 1H), 4.27 (m, 1H), 3.82 (m, 1H), 3.70 (s, 6H), 2.61–2.87 (m, 2H), 1.38 (s, 9H), 1.35 (s, 3H), 1.18 (s, 3H). ^13^C NMR: *δ* 157.73, 156.38, 155.55, 150.95, 149.23, 145.08, 137.22, 136.97, 136.74, 129.67, 128.24, 127.72, 126.54, 117.58, 113.26, 113.03, 112.99, 88.68, 82.41, 81.33, 80.38, 77.91, 69.39, 54.96, 41.85, 28.17, 27.16, 25.57. MS (ESI^+^): *m/z* [M+H]^+^ calcd for C_39_H_45_N_6_O_8_, 725.33; found, 725.32.

### 4.37 5′-(*tert*-Butyloxycarbamoyl)-5′-deoxy*-N*^2^-(4,4′-dimethoxytrityl)-*N*^7^-methyl-2′,3′-*O*-(1-methylethylidene)guanosine (**9a**)

Using general method 1 from **8** and methyl iodide. Yellow powder (98%). Mp 106–107 °C. ^1^H NMR: *δ* 11.48 (s, 1H, NH), 9.20 (s, 1H), 8.07 (s, 1H) 7.15–7.34 (m, 9H), 6.89 (m, 1H), 6.86 (d, *J* = 8.9 Hz, 4H), 5.45 (d, *J* = 2.1 Hz, 1H), 4.82 (dd, *J*_1_ = 6.1 Hz, *J*_2_ = 2.1 Hz, 1H), 4.39 (dd, *J*_1_ = 6.1 Hz, *J*_2_ = 1.4 Hz, 1H), 4.08 (m, 1H), 3.93 (m, 3H), 3.71 (s, 6H), 2.72–3.03 (m, 2H), 1.38 (s, 9H), 1.35 (s, 12H), 1.10 (s, 3H). ^13^C NMR: *δ* 157.89, 155.63, 152.80, 147.19, 144.32, 137.82, 136.10, 136.03, 129.74, 129.66, 128.26, 127.81, 126.81, 113.07, 112.75, 108.54, 92.19, 86.13, 82.00, 81.33, 78.18, 69.81, 55.02, 41.63, 35.62, 28.11, 26.61, 25.14. MS (ESI^+^): *m/z* [M]^+^ calcd for C_40_H_47_N_6_O_8_, 739.34; found, 739.34.

### 4.38 *N*^7^-Benzyl-5′-(*tert*-butyloxycarbamoyl)-5′-deoxy*-N*^2^-(4,4′-dimethoxytrityl)-2′,3′-*O*-(1-methylethylidene)guanosine (**9b**)

Using general method 1 from **8** and benzyl bromide. The crude product was purified by SiO_2_ column chromatography (CHCl_3_—MeOH, 9:1) to afford the title compound as a white powder (73%). ^1^H NMR ((CD_3_)_2_CO): *δ* 10.11 (s, 1H), 7.02–7.67 (m, 15H), 6.66–6.94 (m, 4H), 6.45–6.65 (t, *J* = 5.9 Hz, 1H), 5.42–5.77 (m, 3H), 5.12 (m, 1H), 4.72 (m, 1H), 4.22–4.44 (m, 1H), 3.67 (s, 6H), 3.14–3.48 (m, 2H), 1.43 (s, 3H), 1.40 (s, 9H), 1.17 (s, 3H). ^13^C NMR ((CD_3_)_2_CO): *δ* 158.99, 157.32, 157.10, 149.16, 146.44, 138.26, 138.18, 136.48, 135.60, 131.44, 131.37, 130.02, 129.57, 129.46, 128.16, 127.08, 114.08, 113.42, 108.70, 93.82, 89.03, 84.51, 82.73, 79.29, 71.32, 55.37, 52.32, 43.47, 34.40, 28.63, 27.53, 26.03. MS (ESI^+^): *m/z* [M]^+^ calcd for C_46_H_51_N_6_O_8_, 815.4; found, 815.3.

### 4.39 5′-Amino-5′-deoxy-*N*^7^-methylguanosine (**10a**)

Using general method 3 from **9a**. After completion of the reaction the solvent was evaporated and the residue was suspended in H_2_O and extracted with CHCl_3_. The combined extracts were dried (MgSO_4_) and evaporated. The residue was purified by preparative HPLC using a Luna HILIC column (*t*_R_ 8.0 min; gradient from 0% A to 100% A over 12.5 min, where A is 5:4:1 MeCN—H_2_O–50 mM aq NH_4_HCOO pH 3.2, and B is 9:1 MeCN–50mM aq NH_4_HCOO pH 3.2) to afford the title compound as a white solid (51%). ^1^H NMR: *δ* 9.32 (s, 1H), 8.26 (s, 2H), 6.99 (br s, 2H), 5.82 (dd, *J* = 4.7 Hz, 1H), 4.47 (t, *J* = 4.7, 1H), 4.18 (t, *J* = 4.7 Hz, 1H), 4.18 (t, *J* = 4.7 Hz, 1H), 4.04 (m, 1H), 4.00 (s, 3H), 3.41 (m, 2H). ^13^C NMR: *δ* 158.68, 156.83, 149.26, 135.39, 107.69, 89.51, 82.52, 73.43, 70.57, 40.94, 35.46. MS (ESI^+^): *m/z* [M]^+^ calcd for C_11_H_17_N_6_O_4_, 297.13; found, 297.13.

### 4.40 5′-Amino-*N*^7^-benzyl-5′-deoxyguanosine (**10b**)

Using general method 3 from **9b**. The crude product was triturated with Me_2_CO and the colourless precipitate was filtered and dried (44%). ^1^H NMR: *δ* 8.50 (s, 1H), 7.51–7.61 (m, 2H), 7.32–7.43 (m, 3H), 5.98 (d, *J* = 4.4 Hz, 2H), 5.70 (s, 2H), 4.87 (t, *J* = 4.4 Hz, 1H), 4.38 (m, 1H), 4.32 (m, 1H), 3.36–3.64 (m, 2H). ^13^C NMR: *δ* 157.18, 154.77, 149.61, 136.66, 134.65, 128.90, 128.77, 128.38, 106.99, 90.31, 81.50, 73.10, 70.85, 51.40, 40.80. Anal. RP-HPLC: *t*_R_ 3.51 min (method 1, >95%). MS (ESI^+^): *m/z* [M]^+^ calcd for C_17_H_21_N_6_O_4_, 373.2; found, 373.2.

### 4.41 5′-Deoxy*-N*^7^-methyl-5′-((1*H*-tetrazol-5-yl)methylcarbamoyl)guanosine (**11a**)

To a stirred solution of **10a** (15 mg, 0.05 mmol) in DMF (1 mL) were added Et_3_N (8 μL, 0.05 mmol), *N*,*N*′-diisopropylcarbodiimide (8 μL, 0.05 mmol), and 1*H*-tetrazole-5-acetic acid (7 mg, 0.05 mmol). The mixture was stirred for 24 h at r.t. and diluted with Me_2_CO (10 mL). The precipitate was collected, dried, and purified by preparative HPLC using a Luna HILIC column (gradient from 0% A to 100% A over 12.5 min, where A is 5:4:1 MeCN—H_2_O–50 mM aq NH_4_HCOO pH 3.2, and B is 9:1 MeCN–50mM aq NH_4_HCOO pH 3.2) to afford the title compound as a white solid (3 mg, 14%). ^1^H NMR (D_2_O): *δ* 8.26 (s, 1H), 5.77 (d, *J* = 4.3 Hz, 1H), 4.72 (t, *J* = 4.3 Hz, 1H), 4.51 (m, 2H), 4.09 (m, 2H), 3.88 (s, 3H), 3.76 (s, 2H), 3.47 (m, 2H). ^13^C NMR (D_2_O): *δ* 171.20, 163.14, 162.86, 155.40, 155.08, 149.44, 108.68, 90.23, 83.33, 73.67, 70.69, 40.39, 35.79, 31.89. Anal. RP-HPLC: *t*_R_ 0.67 min (method 1: >95%), *t*_R_ 0.33 min, (method 2, >95%.) MS (ESI^+^): *m/z* [M^+^] calcd for C_14_H_19_N_10_O_5_, 407.15; found, 407.16.

### 4.42 *N*^7^-Benzyl-5′-deoxy*-*5′-((1*H*-tetrazol-5-yl)methylcarbamoyl)guanosine (**11b**)

This compound was obtained from **10b** analogously to preparation and purification of 11a (34%). ^1^H NMR: *δ* 10.19 (s, 1H), 8.38 (t, *J* = 5.6 Hz, 1H), 8.31 (s, 2H), 7.74 (s, 1H), 7.49–7.58 (m, 2H), 7.21–7.41 (m, 3H), 5.85 (d, *J* = 5.3 Hz, 1H), 5.75 (dd, *J*_1_ = 20.0 Hz, *J*_2_ = 15.0 Hz, 1H), 4.40 (t, *J* = 5.2 Hz, 1H), 4.06 (m, 2H), 3.34–3.67 (m, 4H). ^13^C NMR: *δ* 169.95, 165.46, 158.39, 155.95, 150.63, 136.32, 135.42, 129.19, 129.00, 128.92, 107.00, 88.05, 84.92, 74.52, 70.80, 51.77, 40.90, 34.05. Anal. RP-HPLC: *t*_R_ 1.40 min (method 1, >95%), *t*_R_ 0.77 min (method 2, >95%). MS (ESI^+^): *m/z* [M^+^] calcd for C_20_H_23_N_10_O_5_, 483.18; found, 483.15.

### 4.43 Virtual screening

An eIF4E–Bn^7^GMP complex crystal structure (PDB code 2V8X) [15] was used for docking purposes, and docking was based on the lead-like subset of compounds in the ZINC database [40]. Receptor preparation was performed using the Biopolymer function in SYBYL 8.0 (Tripos International, 1699 South Hanley Rd., St. Louis, MO, 63144, USA). The entire binding site occupied by Bn^7^GMP in the eIF4E template structure was defined as the docking site. Ligand 3D co-ordinates were generated using CONCORD (R. S. Pearlman, “Concord,” distributed by Tripos International, St. Louis, MO, 63144, USA). Conformer (up to 200 per compound) generation (OMEGA version 2.4.6) [54], [55], as well as docking and scoring (FRED module of the OEDocking version 3.0.0) [41], [42], were carried out using OpenEye Scientific Software (Santa Fe, NM, USA; http://www.eyesopen.com). Scoring of docked poses was performed using a combination of the OEDocking scoring functions Chemgauss3 and Zapbind (using Poisson-Boltzman electrostatics approximation), together with a semi-empirical method using the LocalSCFv2.0 application (Fujitsu FQS Poland Sp. z o. o., Krakow, Poland) [56]. Virtual hit compounds were acquired from vendors (Table 1) and were characterised by LC-MS. Two different batches of compound **14** with slightly different appearance and aqueous solubility were obtained. However, these were indistinguishable by LC-MS and NMR, and spectral data was in accord with the structure shown.

### 4.44 Western blotting

SDS-PAGE and electroblotting were carried out as usual. For Western blotting, the following antibodies were used: rabbit anti-BCL, Cell Signalling #2872; mouse anti c-Myc (9E10), SantaCruz #sc-040; rabbit anti-GAPDH, Cell Signalling #2118; rabbit anti-eIF4E-BP1, Cell Signalling #9644; rabbit anti-phospho-eIF4E-BP1, Cell Signalling #9459; rabbit anti-eIF4E, Cell Signalling #9742; rabbit anti-eIF2A (D7D2) XP, Cell Signalling #5324; mouse anti-eIF2A, Cell Signalling #2103; rabbit anti-cyclin D1, SantaCruz #753; mouse anti-β-actin, Sigma #A3854; (primary); anti-mouse-HRP conjugate, 1:5,000, Dako P0447; anti-rabbit-HRP conjugate, 1:10,000, GE Healthcare NA9340V.

### 4.45 Recombinant eIF4E

Expression and purification of eIF4E followed a modified procedure of that already published [15]. Briefly, the procedure was modified to allow for rapid, scale-up production of eIF4E and will be published separately (manuscript in preparation). Following purification, the protein concentration was adjusted to 1 mg mL^−1^ (Vivaspin concentrator, 5 kDa cut-off) and the solution was flash-frozen in 200-μL aliquots, which were stored at −80 °C.

### 4.46 Fluorescence polarisation assay

A fluorescent probe (EDA-m^7^GDP–ATTO-465; NU-827-465) was obtained from Jena Biosciences; 96-well black plates (Optiplate^®^) were from Perkin Elmer. Recombinant eIF4E and the probe were diluted (20 mM HEPES, pH 7.4, 100 mM KCl, 1 mM DTT) to obtain the required concentration. m^7^GTP (Sigma-Aldrich) and m^7^GMP were used as positive controls. The final volume in each well was 100 μL. Compound screening was performed using a final DMSO concentration of 1%. Fluorescence polarisation measurements were recorded on a Perkin-Elmer Envision 2104 plate reader. For *K*_d_ calculation, logarithmic serial dilutions of test compounds were prepared. Background-corrected readings were plotted against test compound concentration in GaphPad Prism (Version 5.0) in order to obtain *K*_d_ values.

### 4.47 Crystallisation of ligand-bound eIF4E

A 3.2-mM solution of 4G-I (eIF4G-derived peptide, KKRYDREFLLGFQF) or 4EBP-I (4E-BP-derived peptide, RIIYDRKFLMECRN) in crystallisation buffer (50 mM Tris, pH 8.0, 150 mM KCl, 1 mM DTT) was added to an equal volume of a solution of recombinant human eIF4E (8 mg mL^−1^). A ligand solution (50 or 100 mM) in DMSO was added to a final DMSO concentration of 2.5%. Thin needle-like crystals grew over a period of 3–7 d by the hanging drop method (1:1 protein–mother liquor) over 21–31% PEG-8,000, 1–3% (NH_4_)_2_SO_4_, 100 mM HEPES, pH 7.5. Drops containing crystals were added to 50 μL mother liquor vortex-mixed for 3 min. This was used as a seed stock (at 1:1 and 1:10 dilution) for seeding back into the same conditions. Needle-like crystals grew in 1–7 d and were frozen directly in liquid N_2_. For back-soaking experiments with ligand **7a** complex crystals, crystals were transferred to a drop containing a 1:1 ratio of crystallisation buffer and 100 mM HEPES, pH 7.5, 21% PEG-8,000, supplemented with **7a**. The drop was equilibrated over 100 mM HEPES, pH 7.5, 21% PEG-8000 for 5 min before flash-freezing the crystals in liquid N_3_.

X-ray crystallography data collection and structure refinement. Data were collected at Diamond Light Source, UK, from single crystals using beamlines I02, I04, and I04-1, at 100 K, and were processed using either iMOSFLM [57] or XDS [58] using scaling with SCALA [59]. The structures were solved with the AutoMR command in the PHENIX suite [60], which uses the programme PHASER [61]. A single eIF4E and 4E-BP chain from the unit cell of PDB entry 2V8Y (ligand removed) was used as the search model and all structures were subjected to several rounds of refinement in PHENIX and manual refinement in COOT [62], including ligand fitting and a rebuilding of the 4G-I peptide chain (where necessary).

### 4.48 Rabbit reticulocyte lysate *in vitro* translation assays

PCR (hot KOD polymerase kit, Novagen^®^; Qiaquick PCR purification kit, QIAGEN) was used to amplify the luciferase DNA sequence from a pGl3 vector with the primers GCGGCCGGCCGCCCCGACTCTAGAA and GGGGTAATACGACTCACTATAGGGTCC-ACCTCGAATCACTAGTCAGCTG. Alternatively, the plasmid pRHCVF, which contains *Renilla* luciferase as the upstream cistron and firefly luciferase as the downstream cistron preceded by the HCV IRES, was digested with *Hpa*I and use to prime *in vitro* transcription reactions. *In vitro* RNA synthesis was performed on the resulting DNA using a mMessage mMachine^®^ T7 kit (Ambion). The resulting capped RNAs were purified using microSpin G-50 columns (GE Healthcare), analysed by agarose gel electrophoresis, and were stored in aliquots at −80 °C. An *in-vitro* translation Rabbit Reticulocyte Lysate System kit (Promega) was used as per the manufacturer's instructions. Briefly, 20 ng capped RNA was incubated with rabbit reticulocyte lysate in a final reaction volume of 20 μL. The mixture was incubated at 30 °C for 90 min and then placed on ice for immediate analysis. Diluent (H_2_O) was replaced by test compound solutions in the course of compound screening and the final DMSO concentration was limited to 1%. Assays were performed with m^7^GTP as a positive control and a DMSO negative control (which was assigned a 100% value).

### 4.49 Ultraviolet cross-linking assay

In a way similar to RNA luciferase synthesis, a 392-base coding region of the pGL3 vector was amplified and the corresponding RNA was generated using the T7 RiboMax large-scale production system (Promega) according to the manufacturer's instructions. The resulting transcript was then cap-radiolabelled with ^32^P-[α-GTP] (specific activity 400 Ci mmol^−1^, Hartmann Analytic GmbH) using a vaccinia virus capping system (New England Biolabs) and purified using a microSpin G-50 column. A 1-μL aliquot of the labelled RNA was transferred to a scintillation vial with 2 mL scintillation liquid (Ecoscint A, National Diagnostics), and was read in a scintillation counter (Wallac Winspectral 1414 liquid scintillation counter). The disintegration per minute readings were converted to Ci units, and the concentration of radiolabelled capped RNA was then calculated based the original ^32^P-GTP stock concentration after correction based on decay time. Assay procedures: to work out suitable concentrations of RNA and eIF4E to be used in the assay, a titration experiment of eIF4E against a constant concentration of labelled RNA was carried out. The assay was performed using 96-well flat-bottom plates (Corning, Costar^®^), in a volume of 20 μL for each reaction (in a ratio of 10:5:5 for buffer, RNA, eIF4E, respectively). Plates were kept on ice and were exposed to a UV radiation source of 275 J m^2^ at a distance of 3 cm for a period of 30 min. The plate wells were then treated with a mixture of RNase (0.1 mg mL^−1^ RNase A, 0.1 mg mL^−1^ RNase T1, 0.1 mg mL^−1^ RNase V1) at 37 °C for 30 min. Samples were then separated by SDS-PAGE and the gels were dried and exposed to autoradiography film for 24 h, before being read using a Typhoon 9500 FLA scanner. Band intensities were quantified using image analysis software (Image J, Version 1.46) relative to a DMSO controls.

### 4.50 ^35^S-methionine incorporation assay

HeLa cells were seeded into 12-well plates at 1 × 10^5^ cells per well for 2-h and at 6 × 10^4^ cells per well for 24-h treatment experiments. Cells were incubated for 24 h until they reached 70% confluency and were then treated with test compounds. Cells were methionine-starved for 30 min by changing to a Met-free DMEM medium, with renewed test compound addition. A 1.5-μL aliquot of ^35^S-methionine (30 μCi mL^−1^, Hartmann Analytic) was added to each well of the plates, which were incubated for 30 min. The plates were placed on ice and medium was removed. The cells were washed with warm phosphate-buffered saline (PBS) twice and lysed by storing at −80 °C for 30 min in lysis buffer (Promega). For quantification, protein was precipitated with trichloroacetic acid, collected, dried, transferred to scintillation liquid, and read in a scintillation counter.

### 4.51 Polysome profiling assay

HeLa cells were seeded in 15-cm diameter dishes and were left for 24 h to reach 70% confluency. Cells were treated with test compound for 2 h and cycloheximide (0.1 mg mL^−1^) was added. Cells were harvested on ice and then lysed using lysis buffer 300 mM NaCl, 15 mM MgCl_2_, 15 mM Tris (pH 7.5) containing 1 mg mL^−1^ heparin sulfate and 0.1 mg mL^−1^ cycloheximide, supplemented with 0.1% (v/v) Triton X-100. Cell lysates were loaded on sucrose gradients (10–60% sucrose in 12-mL Sorvall centrifuge tubes) made with the same buffer, and were ultracentrifuged using a SW40Ti (Beckman Coulter) centrifuge at 38,000 r.p.m. for 2 h. The gradients were then separated into fractions of 1 mL with continuous UV absorbance recording.

## Author contributions

The manuscript was written through contributions of all authors. All authors have given approval to the final version of the manuscript.

## PDB ID codes

5EI3, 5EIR, 5EKV, 5EHC.

## Figures and Tables

**Table 1:** Chemical structures and predicted properties of virtual hits **12**–**16** from structure-based design.


Cmpd	Source	CAS^a^	*M*_r_	Predicted properties^b^						
				logP	logD^c^	TPSA^d^ (Å^2^)	nRot^e^	logS_i_^f^	Drug-like^g^	Lead-like^h^
**12a**	Asinex BAS04026402	557064-00-3	356.4	0.9	−1.1	90	7	−4.7	yes	yes
**12b**	Asinex BAS02054521	338425-77-7	325.4	1.2	1.8	87	5	−4.9	yes	yes
**12c**	Asinex BAS02563297	333412-47-8	325.4	1.2	1.8	87	5	−4.9	yes	yes
**13**	Asinex ASN03777735	459445-48-8	420.6	4.3	4.3	76	4	−5.9	yes	no
**14**	Asinex ASN03775814	461002-66-4	423.5	4.0	4.0	63	6	−7.1	yes	no
**15**	ChemBridge 6373744	426248-94-4	336.8	3.8	3.8	66	3	−6.9	yes	yes
**16**	ChemBridge 8893829	899375-48-5	349.8	2.3	2.3	62	1	−4.5	yes	yes

a Chemical Abstracts Registry Number.

b Using ChemAxon InstantJChem version 15.6.8.0.

c Predicted logD at pH = 7.4.

d Topological polar surface area.

e Number of non-terminal rotatable bonds.

f Log_10_ of predicted molar intrinsic aqueous solubility.

g Criteria: nRot < 10 and TPSA < 140 Å^2^[46].

h Criteria: *M*_r_ ≤ 450, −4 ≤ logD_7.4_ ≤ 4, number of rings ≤ 4, nRot ≤ 10, number of H-bond donors ≤ 5, number of H-bond acceptors ≤ 8 [47].

**Table 2:** Summary of biological activities of compounds **4**–**16**.^a^

Cmpd	FP assay		RNA cross-linking assay		Reticulocyte lysate assay	^35^S-Met incorporation assay	
	Inhibition (%)^b^	*K*_d_ (μM)	Inhibition (%)^b^	IC_50_ (μM)	IC_50_ (μM)	IC_50_ (HeLa)	IC_50_ (MCF-7)
**m**^7^**GTP**	97.3 ± 2.4^c^	0.37 ± 0.03	92.8 ± 1.6^c^	0.72 ± 0.14	8.6 ± 1.1	Inactive	Inactive
**m**^7^**GMP**	86.3 ± 5.0	3.9 ± 0.5	97.5 ± 0.8	1.7 ± 0.1	46.9 ± 3.4	Inactive	Inactive
**4a**	14.4 ± 3.2	n.d.^d^	Inactive	n.d.	n.d.	n.d.	n.d.
**4b**	9.1 ± 5.5	n.d.	Inactive	n.d.	n.d.	n.d.	n.d.
**4c**	15.2 ± 4.7	n.d.	Inactive	n.d.	n.d.	n.d.	n.d.
**4d**	2.0 ± 6.3	n.d.	Inactive	n.d.	n.d.	n.d.	n.d.
**4e**	Inactive	n.d.	Inactive	n.d.	n.d.	n.d.	n.d.
**4f**	13.2 ± 4.2	n.d.	Inactive	n.d.	n.d.	n.d.	n.d.
**4g**	3.9 ± 5.2	n.d.	Inactive	n.d.	n.d.	n.d.	n.d.
**4h**	1.8 ± 7.7	n.d.	6.5 ± 7.1	n.d.	n.d.	n.d.	n.d.
**4i**	Inactive	n.d.	Inactive	n.d.	n.d.	n.d.	n.d.
**7a**	89.0 ± 1.7	56.5 ± 6.9	42.9 ± 4.8	12.6 ± 0.5	65.9 ± 13.6	Inactive	Inactive
**7b**	49.4 ± 6.3	133 ± 28	48.6 ± 1.3	18.7 ± 5.0	165 ± 32	Inactive	Inactive
**7c**	Inactive	n.d.	22.2 ± 1.8	n.d.	n.d.	n.d.	n.d.
**11a**	9.7 ± 2.9	n.d.	25.8 ± 2.9	n.d.	n.d.	n.d.	n.d.
**11b**	30.2 ± 2.8	n.d.	15.3 ± 4.8	n.d.	n.d.	n.d.	n.d.
**12a**	13.6 ± 2.2	n.d.	10.2 ± 3.3	n.d.	n.d.	n.d.	n.d.
**12b**	25.8 ± 5.9	n.d.	Inactive	n.d.	n.d.	n.d.	n.d.
**12c**	8.5 ± 2.8	n.d.	Inactive	n.d.	n.d.	n.d.	n.d.
**13**	Inactive	n.d.	Inactive	n.d.	n.d.	n.d.	n.d.
**14**	68.1 ± 0.8	57.8 ± 13.5	47.5 ± 6.7	38.6 ± 10.6	65.1 ± 12.5	35.2 ± 4.0	37.7 ± 10.4
**15**	Inactive	n.d.	3.5 ± 3.2	n.d.	n.d.	n.d.	n.d.
**16**	Inactive	n.d.	33.8 ± 8.1	n.d.	n.d.	n.d.	n.d.

a All experiments were carried out on three independent occasions.

b At 100 μM concentration unless otherwise indicated.

c At 10 μM concentration.

d Not determined.

**Table 3:** Crystallographic data.

	eIF4E:4G:7**a** (PDB 5EI3)	eIF4E:4G:7**a** back-soak (PDB 5EIR)	eIF4E:4EBP:4**a** (PDB 5EKV)	eIF4E:4G:4**f** PDB 5EHC)
Data collection				
Resolution (Å)	1.71	2.69	3.61	2.40
Space group	*P*2_1_22_1_	*P*2_1_22_1_	*P*2_1_2_1_2_1_	*P*2_1_22_1_
Unit cell (Å)				
*a*	38.6	38.3	38.5	38.5
*b*	52.1	52.2	100.8	52.2
*c*	125.2	123.7	136.5	125.7
Unique reflections^a^	28165 (4039)	6814 (854)	6413 (889)	10425 (1466)
Completeness (%)^a^	100 (100)	93.8 (85.8)	97.8 (95.5)	99.7 (99.7)
R_merge_ (%)^a^	9.4 (34.2)	16.0 (35.9)	28.2 (42.3)	19.2 (33.9)
I/σ^a^	15.7 (5.3)	5.5 (3.0)	4.6 (3.1)	8.9 (5.3)
Refinement				
*R* (%)	16.6	24.8	26.8	21.8
*R*_free_ (%)	18.7	27.2	34.1	26.4
Mean temperature factor *B* (Å^2^)^b^				
eIF4E	14.1	24.7	17.1/20.8^e^	19.4
4G/4EBP	14.0	28.1	17.5/18.8^e^	19.2
Ligand	22.1	38.5 (36.9)^d^	18.4/24.1^e^	42.4
SO_4_	29.5	(46.1)^d^	–	–
Occupancy (%)^b^				
Ligand	1.0	0.52 (0.48)^d^	0.88/0.74^e^	1.0
SO_4_	1.0	0.48	–	–
All atoms used in refinement	2103	1790	3265	1846
Water	355	32	0	139
Validation^c^ (% of all residues)				
Favoured	98.0	99.0	97.8	97.9
Allowed	100.0	100.0	100.0	100.0
Disallowed	0.0	0.0	0.0	0.0

a Values in parentheses refer to the highest-resolution shell.

b *B*-factor and occupancy refinement were performed using Phenix.

c Validation was performed using MolProbity.

d Values in parenthesis refer to the lowest occupancy model.

e Values for both copies in the asymmetric unit.
